# Coping with salinity stress: segmental group 7 chromosome introgressions from halophytic *Thinopyrum* species greatly enhance tolerance of recipient durum wheat

**DOI:** 10.3389/fpls.2024.1378186

**Published:** 2024-04-29

**Authors:** Sana Tounsi, Debora Giorgi, Ljiljana Kuzmanović, Olfa Jrad, Anna Farina, Alessandra Capoccioni, Rayda Ben Ayed, Faiçal Brini, Carla Ceoloni

**Affiliations:** ^1^Biotechnology and Plant Improvement Laboratory, Centre of Biotechnology of Sfax (CBS), University of Sfax, Sfax, Tunisia; ^2^ENEA Casaccia Research Center, Department for Sustainability, Biotechnology and Agroindustry Division, Rome, Italy; ^3^Department of Agriculture and Forest Sciences (DAFNE), University of Tuscia, Viterbo, Italy; ^4^Department of Agronomy and Plant Biotechnology, National Institute of Agronomy of Tunisia (INAT), University of Carthage, Tunis, Tunisia; ^5^Laboratory of Extremophile Plants, Centre of Biotechnology of Borj-Cédria, Hammam-lif, Tunisia

**Keywords:** abiotic stress, alien gene transfer, chromosome engineering, wild wheat relatives, *Thinopyrum ponticum*, *Thinopyrum elongatum*, *Triticum*, sustainability

## Abstract

Increased soil salinization, tightly related to global warming and drought and exacerbated by intensified irrigation supply, implies highly detrimental effects on staple food crops such as wheat. The situation is particularly alarming for durum wheat (DW), better adapted to arid/semi-arid environments yet more sensitive to salt stress than bread wheat (BW). To enhance DW salinity tolerance, we resorted to chromosomally engineered materials with introgressions from allied halophytic *Thinopyrum* species. “Primary” recombinant lines (RLs), having portions of their 7AL arms distally replaced by 7el_1_L *Th. ponticum* segments, and “secondary” RLs, harboring *Th. elongatum* 7EL insertions “nested” into 7el_1_L segments, in addition to near-isogenic lines lacking any alien segment (CLs), cv. Om Rabia (OR) as salt tolerant control, and BW introgression lines with either most of 7el_1_ or the complete 7E chromosome substitution as additional CLs, were subjected to moderate (100 mM) and intense (200 mM) salt (NaCl) stress at early growth stages. The applied stress altered cell cycle progression, determining a general increase of cells in G1 and a reduction in S phase. Assessment of morpho-physiological and biochemical traits overall showed that the presence of *Thinopyrum* spp. segments was associated with considerably increased salinity tolerance versus its absence. For relative water content, Na^+^ accumulation and K^+^ retention in roots and leaves, oxidative stress indicators (malondialdehyde and hydrogen peroxide) and antioxidant enzyme activities, the observed differences between stressed and unstressed RLs versus CLs was of similar magnitude in “primary” and “secondary” types, suggesting that tolerance factors might reside in defined 7el_1_L shared portion(s). Nonetheless, the incremental contribution of 7EL segments emerged in various instances, greatly mitigating the effects of salt stress on root and leaf growth and on the quantity of photosynthetic pigments, boosting accumulation of compatible solutes and minimizing the decrease of a powerful antioxidant like ascorbate. The seemingly synergistic effect of 7el_1_L + 7EL segments/genes made “secondary” RLs able to often exceed cv. OR and equal or better perform than BW lines. Thus, transfer of a suite of genes from halophytic germplasm by use of fine chromosome engineering strategies may well be the way forward to enhance salinity tolerance of glycophytes, even the sensitive DW.

## Introduction

Due to the many environmental components that soil salinization severely affects, it is considered an ecological disaster (Tedeschi, 2020). Whether induced by natural processes and/or anthropogenic activities (principally irrigation), it leads to degradation of soil quality and, in turn, to detrimental effects on all directly or indirectly soil-dependent organisms, including crop plants that represent major food source for mankind, such as wheat (Eynard et al., 2005; Cuevas et al., 2019; El Sabagh et al., 2021; Ullah et al., 2021). Increased salinization, mainly due to excess accumulation of sodium chloride (NaCl), is tightly related to other climate change–driven abiotic stresses. In fact, as a result of global warming and drought, an array of phenomena, from an increase in evaporation and a decrease in precipitation to sea level rise and seawater intrusion into freshwater ecosystems, up to prolonged irrigation supply, poor drainage conditions, and use of marginal water, are concurring to increase the percentage of saline-alkali soils, particularly in arid and semi-arid regions (Cuevas et al., 2019; Tedeschi, 2020; Hopmans et al., 2021; Mazhar et al., 2022; Pearce, 2022; Singh, 2022). Excess salinity currently affects nearly 7% of the world’s total land area, but projections for 2050 indicate as much as 50% of arable land being salinized (Hu and Schmidhalter, 2023), a figure already approached in irrigated soils, providing nearly half of global food production (Bannari and Al-Ali, 2020; Hopmans et al., 2021; Ullah et al., 2021; Singh, 2022). Therefore, if cropping will take place more and more on salt-affected soils, substantial improvements in the salt tolerance of crops are needed. This concern is particularly alarming for wheat (*Triticum* spp.), ranking first in global grain production and representing the staple food for over 36% of world’s population, yet with relatively limited tolerance to high soil salinity levels (Hasanuzzaman et al., 2017; El Sabagh et al., 2021).

Salinity stress significantly impairs all plant growth phases, from seed germination (SG) and seedling establishment to vegetative and reproductive development, ultimately impacting on yield. Excess salinity causes an early osmotic stress and a later ionic toxicity (Munns and Tester, 2008; Hu and Schmidhalter, 2023). Salt-induced osmotic changes in the rootzone lower plant ability to extract water from the soil and accelerate water loss from leaves, reducing turgor and overall disturbing plant water relations and, hence, plant growth. Over time, excess accumulation of Na^+^ and/or Cl^–^ ions in plant tissues compromises ion homeostasis, leading to imbalance of other essential ions and nutrients, altogether hampering fundamental metabolic processes and physiological functions, such as photosynthesis (Hasanuzzaman et al., 2017; Guellim et al., 2019; Arif et al., 2020; El Sabagh et al., 2021; Hu and Schmidhalter, 2023). At the cellular level, in addition to impairing regular cell cycle progression (Qi and Zhang, 2020), excess salinity, as other stresses, triggers a burst in production of reactive oxygen species (ROS), responsible for oxidative stress, leading to lipid peroxidation (e.g., membrane damage), protein degradation (e.g. photosynthetic enzymes), and DNA mutation (Carillo et al., 2011; Hasanuzzaman et al., 2017).

Along with the ability to reduce the ionic stress by minimizing the Na^+^ cytosolic amount, particularly in transpiring leaf tissue, and limiting the K^+^ efflux (Wu et al., 2018b), ROS scavenging represents an essential mechanism of plant salt tolerance (Hasanuzzaman et al., 2020). ROS, which also work as important signal transduction molecules integrated in various stress response pathways, are normally kept at basal non-toxic levels by an array of antioxidative mechanisms (Mittler, 2017; Hasanuzzaman et al., 2020). As these mechanisms become less efficient under stress conditions, a complex system of enzymatic and non-enzymatic reactions and components is triggered to maintain the cellular redox homeostasis (Feki et al., 2017; Hasanuzzaman et al., 2020). Superoxide dismutase (SOD), catalase (CAT), peroxidase (POD), ascorbate peroxidase (APX), glutathione reductase, and glutathione peroxidase are among the most frequently activated ROS-detoxifying enzymes (e.g. Hasanuzzaman et al., 2017; Tounsi et al., 2023). As for non-enzymatic antioxidant molecules, ascorbic acid (AsA) and glutathione (GSH), belonging to the AsA–GSH pathway, play a vital and universal role, along with α-tocopherol (vitamin E), flavonoids, anthocyanins, phenolic compounds, and carotenoids (Foyer and Noctor, 2011; Suzuki et al., 2012; Venkatesh and Park, 2014; Hasanuzzaman et al., 2019, 2020; El Sabagh et al., 2021). Small organic molecules referred to as osmoprotectants or osmolytes are additional fundamental players in the plant response to salinity stress. Certain amino acids, particularly proline, ammonium compounds, sugars, and sugar alcohols, are effective protectants that act not only as compatible solutes in osmotic adjustment but also in maintenance of ion homeostasis, ROS scavenging, modulation of antioxidant enzyme activities, and stabilization of cell structures and of key metabolisms (Hasanuzzaman et al., 2017; El Sabagh et al., 2021).

Plant species vary widely in their response to salinity stress, with osmotic and ion effects being interconnected and often collectively impacting on the plant (Hopmans et al., 2021). Most crop plants, including cereals, are glycophytes. Among them, barley shows the highest ability to withstand salt stress, followed by wheat and then rice (Munns et al., 2006; Roy et al., 2014; Hopmans et al., 2021). Durum wheat (*Triticum durum* Desf., 2*n* = 4*x* = 28, genome AB; DW) is one of the most important crops cultivated in countries surrounding the Mediterranean basin (Xynias et al., 2020). Geographical and environmental/climatic characteristics of the Mediterranean area make it particularly prone to salinization surge: a rapid decay of freshwater resources is registered here, along with temperatures rising faster than the global average and a 25%–30% decline in rainfall predicted by 2080 (Borghini et al., 2014; Cannon, 2022; Pearce, 2022). DW is more adapted to arid and semi-arid conditions compared with bread wheat (*Triticum aestivum* L., 2*n* = 6*x* = 42, genome ABD; BW), yet more sensitive than the latter to excess soil salinity (Yousfi et al., 2010; Carillo et al., 2011; Annunziata et al., 2017). Distinctive traits between the two wheat species include a superior ability to maintain lower Na^+^ accumulation and a higher K^+^/Na^+^ ratio in the root and leaf/shoot tissues manifested by BW (Colmer et al., 2006; Wu et al., 2014; Wu et al., 2018a, Wu et al., 2018b), along with an overall higher expression of salt-responsive homoeologous genes located on the D-subgenome (Yang et al., 2014; Zhang et al., 2016). To enhance DW salinity tolerance via transfer of BW homoeoloci, DW chromosome 4B was engineered with a BW chromosome 4D introgression containing the *Knal* gene for K^+^/Na^+^ discrimination (Dvořák et al., 1994; Luo et al., 1996).

Although with intra-specific variation, marked salinity tolerance characteristics are present in closely related wild Triticeae species, including diploid A genome species (*T. urartu*, *T. monococcum*; see, e.g., Munns et al., 2012; Tounsi et al., 2016) and various diploid or polyploid *Aegilops* species (e.g. Colmer et al., 2006; Farooq and Azam, 2007; Inbart-Pompan et al., 2013; Darko et al., 2020; Pour-Aboughadareh et al., 2021). However, it is within perennial wheatgrasses, belonging the wheat tertiary gene pool, that the greatest number of highly salt tolerant or even truly halophytic Triticeae species can be found, notably those belonging to the *Thinopyrum* genus (Colmer et al., 2006; Flowers and Colmer, 2008). Such species, carriers of many beneficial traits, rarely or not represented in cultivated wheat or closely allied gene pools (Ceoloni et al., 2015), were since long identified as among the few representatives of the Triticeae tribe occupying specialized ecological niches with high salinity features (Dewey, 1960; McGuire and Dvořák, 1981; Dvořák et al., 1988; Farooq, 2009; Ceoloni et al., 2015; Arzani and Ashraf, 2016; Mujeeb-Kazi et al., 2019). They are indigenous of southern Europe, western Asia, and northern Africa, where frequently occur on saline meadows and seashores (e.g., Monsen et al., 2004). Moreover, they were widely naturalized and exploited in disturbed wastelands throughout North America (Scheinost et al., 2008), as well as in Argentina, some European countries, and China (Li et al., 2023 and references therein). Remarkable adaptive abilities in critical environments, such as saline and sodic soils, are exhibited by the decaploid *Thinopyrum ponticum* (2*n* = 10*x* = 70). From BW-*Th. ponticum* hybrids and amphiploids, introgression of single (Yuan and Tomita, 2015) or multiple (Chen et al., 2004) chromosomal segments from the wild parent was obtained, which conferred several major salt tolerance traits to BW. In neither case, however, were the *Th. ponticum* segments assigned to corresponding chromosomes. This was, instead, the case for another salt tolerant *Thinopyrum* species, that is, the diploid *Th. elongatum* (2*n* = 2*x* = 14). From single chromosome addition and substitution lines into BW cv. Chinese Spring (CS), the enhanced salt tolerance exhibited by the CS/*Th. elongatum* amphiploid was found to be controlled by additively acting genes located on many *Th. elongatum* chromosomes, 3E and 7E containing the major determinants of the improved performance of the recipient BW (Dvořák et al., 1988; Omielan et al., 1991; Deal et al., 1999; Zeng et al., 2023). Several recombinant lines (RLs) carrying 3E introgressions on 3A or 3D were obtained (Mullan et al., 2009), and their effects upon salt stress application were analyzed (Deal et al., 1999; Mullan et al., 2009; Zeng et al., 2023). On the other hand, little knowledge is available on the contribution of 7E, of which both arms seem to contribute to the enhancement of the K^+^/Na^+^ ratio in BW, but with a more significant impact of the long arm, that is, 7EL (Deal et al., 1999).

The opportunity to analyze the effects upon salt stress exposure of introgression of group 7 long arms of *Thinopyrum* spp. chromosomes, specifically 7EL from *Th. elongatum* and 7el_1_L (formerly 7AgL) from *Th. ponticum*, has been offered by DW-*Thinopyrum* spp. RLs, developed through chromosome engineering strategies and carrying small distal portions of either 7el_1_L alone (Ceoloni et al., 2005) or with a “nested” 7EL insertion into the respective 7el_1_L segment (Kuzmanović et al., 2019). The *Thinopyrum* spp. introgressions provide the RLs with several valuable attributes, from resistance to wheat diseases, quality- and yield-related traits (Kuzmanović et al., 2014; Ceoloni et al., 2015; Kuzmanović et al., 2016, 2018, 2019, Rossini et al., 2020; Kuzmanović et al., 2021; Fanelli et al., 2023), to enhanced tolerance to heat and combined heat and water deficit stress (Giovenali et al., 2023). However, they were not tested so far for their response to salt stress. Therefore, in view of exploiting such materials in DW sustainable and stress-responsive breeding, this study aimed at assessing the behavior of primary and secondary DW-*Thinopyrum* spp. RLs, along with specific BW-*Thinopyrum* spp. introgression and other control lines (CLs), when exposed to salt (NaCl) stress under controlled conditions. Several morpho-physiological, cellular, and biochemical parameters were evaluated. The analysis revealed an overall highly positive impact of *Thinopyrum* spp. introgressions on DW performance toward salt stress and highlighted specific/major contributions to given tolerance traits of defined alien chromosome portions.

## Materials and methods

### Plant materials

**Figure 1:**
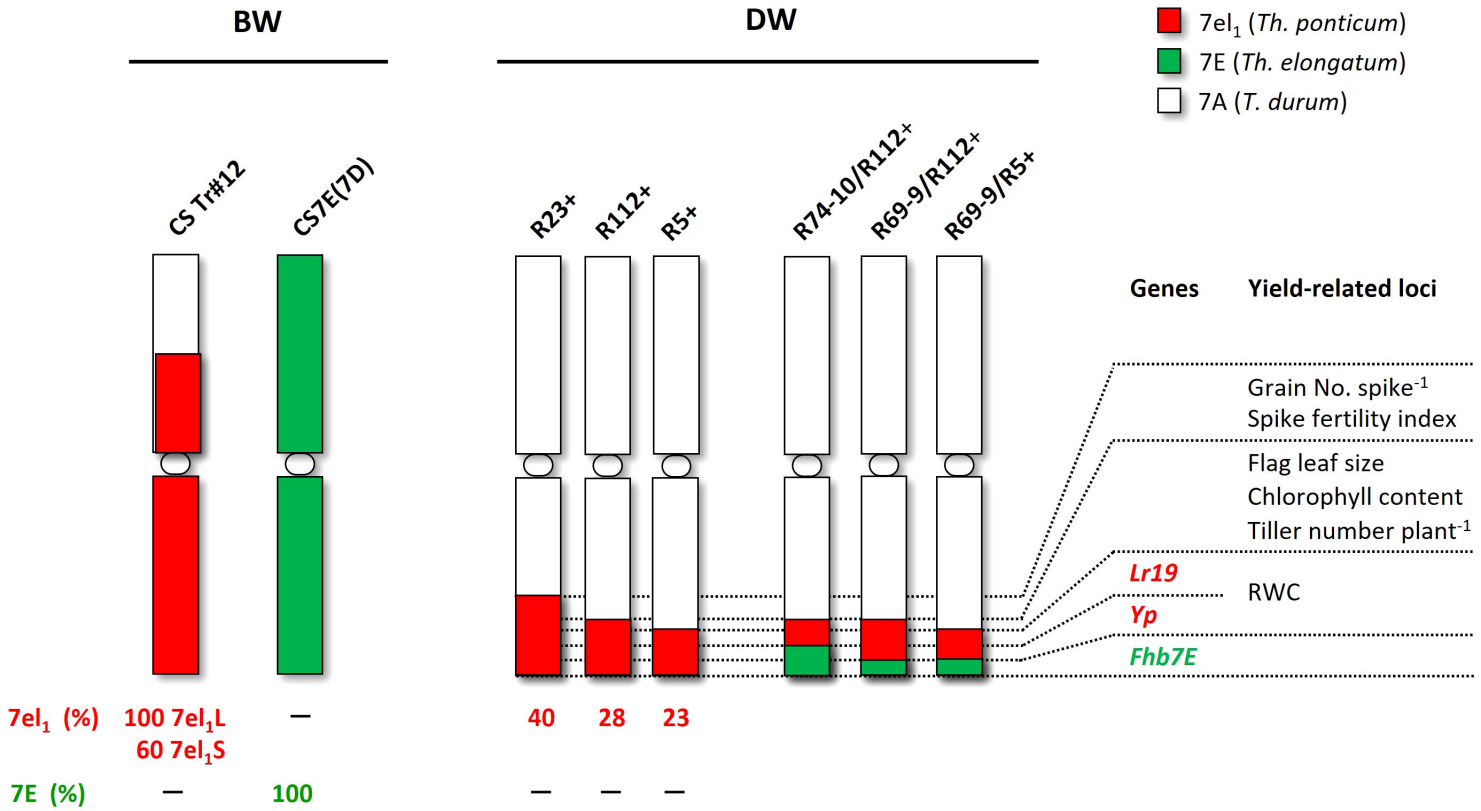
Wheat-*Thinopyrum* spp. lines tested for salt stress tolerance. Six are durum wheat (DW) recombinant lines (RLs), including R5+, R112+, and R23+ (*Th. ponticum* introgressions) and R69-9/R5+, R69-9/R112+, and R74-10/R112+ (*Th. ponticum* + *Th. elongatum* introgressions), and three bread wheat (BW) lines, namely cv. Chinese Spring (CS), the CSTr#12 RL, and the CS7E(7D) substitution line. Relative physical sizes of *Th. ponticum* segments are on scale in the respective 7A chromosomes, whereas for the 7E *Th. elongatum* introgressions, nested into the 7el_1_ *Th. ponticum* segments of the DW recombinants, their relative size versus the hosting segment is roughly deduced from genetic map data (see text).

Six DW-*Thinopyrum* spp. RLs represented the target materials for the salinity assays. They were developed at the University of Tuscia, Viterbo (Italy) and consist of near-isogenic RLs, obtained through chromosome engineering methodologies and repeated backcrosses (BC_4-5_) of the original RLs with the recurrent Italian DW cv. Simeto, followed by several self-generations. RLs included three “primary” types (Ceoloni et al., 2005), carrying a distal *Th. ponticum* 7el_1_L segment occupying a different percentage of the recipient DW 7AL arm, namely R5+ (23%), R112+ (28%), and R23+ (40%). Also employed were “secondary” RLs, harboring a small *Th. elongatum* 7EL insertion “nested” in the most telomeric portion of the *Th. ponticum* segment of R5+ and R112+; these were named R69-9/R5+, R69-9/R112+, and R74-10/R112+, with the R74-10 7EL fraction resulted genetically longer than the R69-9 one (Ceoloni et al., 2017; Kuzmanović et al., 2019), yet both segments being of undefined physical length (**Figure 1**). Together with the “+” RLs, which are homozygous carriers of the given *Thinopyrum* segment, corresponding sib lines, non-carrier of the given alien segment, and, hence, with a “–” symbol accompanying their designations, were also employed in the analyses as CLs. The Tunisian cv. OR was included as a salt-tolerant reference genotype (Brini et al., 2009). Seeds of OR were supplied by INRAT (Institut National de la Recherche Agronomique de Tunisie).

BW lines, all sharing the cv. CS background, were also part of the materials assayed (**Figure 1**). They were as follows: (1) CSTr#12, whose chromosome 7A is mostly replaced by *Th. ponticum* chromosome 7el_1_ (formerly 7Ag; Eizenga, 1987; Ceoloni et al., 1996), employed as donor line of the 7el_1_L segments to the DW recombinants described above; (2) CS7E(7D), whose chromosome 7D is substituted by a complete *Th. elongatum* 7E, used in the chromosome engineering work to develop the “nested” recombinants (Ceoloni et al., 2017; Kuzmanović et al., 2019); (3) CS, working as control of the two previous lines. Seeds of CS and CSTr#12 were originally provided to C.C. by Prof. E.R. Sears, University of Missouri, Columbia, MO (USA), and those of the CS7E(7D) substitution line by Prof. Moshe Feldman, Weizmann Institute of Science, Rehovot (Israel).

### Germination assays and morphological measurements at early stages

For a first assay, 30 seeds of each genotype were surface sterilized and placed in triplicates on Petri dishes on two sheets of wet filter paper with 0 mM NaCl, 100 mM NaCl, or 200 mM NaCl solution and then kept in a growth chamber for 3 days until germination. Germination ability was calculated at day 3 as percentage number of germinated seeds/total number of seeds.

For a second assay, which was repeated twice, a subset of DW RLs, namely, R112+ and R74-10/112+, together with their CLs, R112–, and R74-10/112–, were analyzed. Thirty seeds per genotype and salt concentration were grown in Petri dishes in 0 mM NaCl, 100 mM NaCl, or 200 mM NaCl. At 4 and 10 days after sowing (DAS) the following morphological traits were measured: root length 4 DAS (RL4, mm), root length 10 DAS (RL10, mm), leaf length 4 DAS (LL4, cm), and leaf length 10 DAS (LL10, cm). At 10 DAS, leaf dry weight (LDW, g) and root dry weight (RDW, g) were also measured. RDW was determined on roots pooled from five plantlets, due to the very low root weight after drying. Root and leaf length were measured using ImageJ software (https://www.imagej.nih.gov/ij/download.html).

### Growth conditions and salt treatment in the hydroponics assay

**Figure 2:**
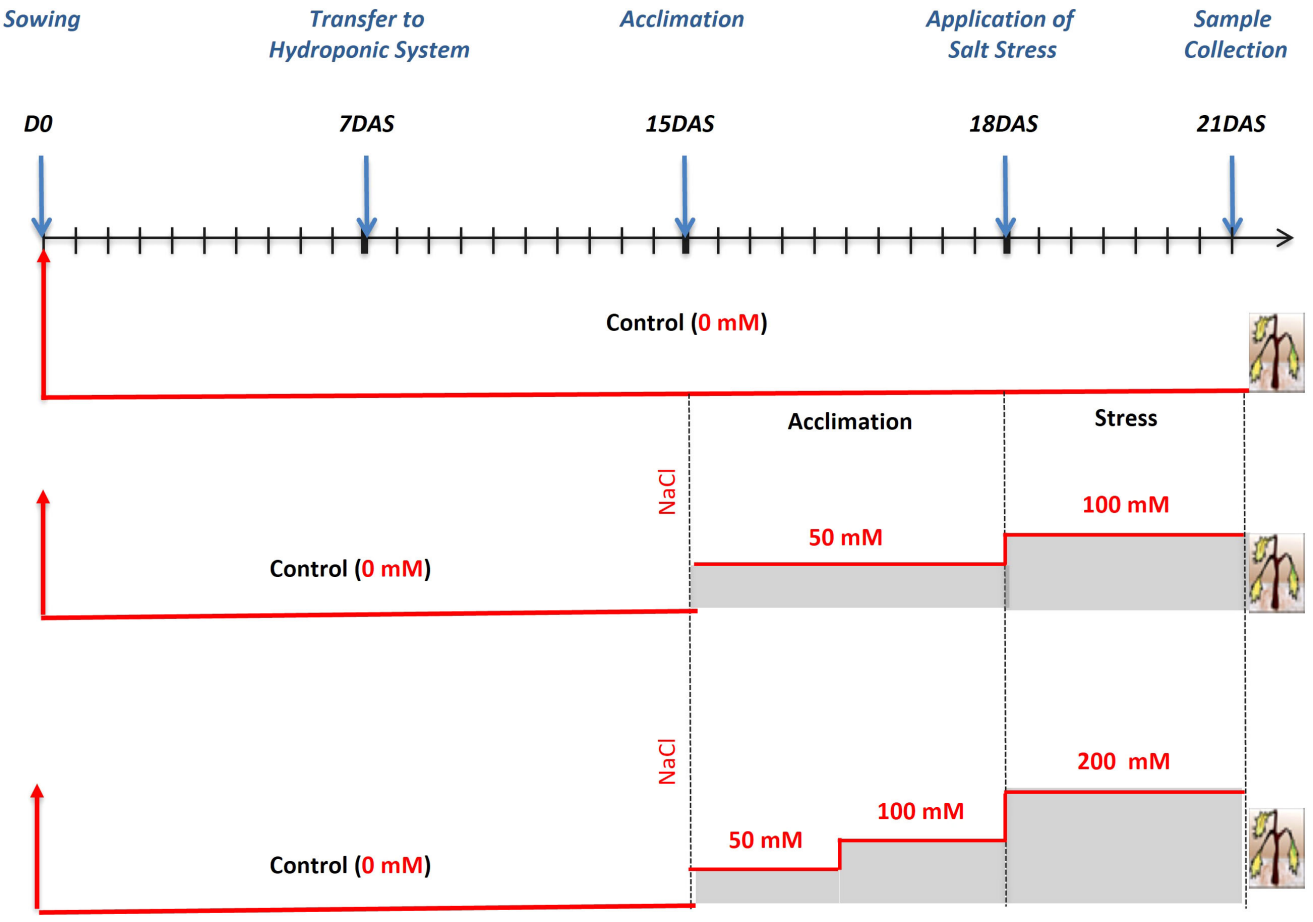
Schematic representation of salt stress application. DAS, days after sowing.

Seeds were surface sterilized with sodium hypochlorite for 15 min, rinsed 3 times with distilled water and then placed in Petri dishes containing Whatman filter paper. The dishes were transferred to a growth chamber at 23°C and 65% relative humidity, with a 16h light/8h dark photoperiod. Seven days after germination, seedlings were transferred to a hydroponic system. The boxes were filled with half-strength Hoagland nutrient solution, which was refreshed every week (Davenport et al., 2005). Each box was constantly aerated by two adjustable air diffusers. After 2 weeks, NaCl concentration was increased progressively for 3 days until reaching 100 mM and 200 mM (**Figure 2**). Control plants were kept in nutrient solution. Plant tissues (roots and leaves) were harvested after 3 days of salt treatment (i.e., on the 21st day after sowing, DAS; **Figure 2**) and used for morpho-physiological and biochemical analyses. For all traits, measurements were performed using at least three biological and three technical replicates. Whole, fully expanded leaves were used for measurements of relative water content (RWC) and leaf surface area (LSA). In the latter case, the third leaf of representative plants of each genotype was consistently employed. The remaining leaves were harvested and used for biochemical analyses. Root elongation (RE) was measured on the main seminal roots and the total biomass used for ion determination.

### Morpho-physiological and biochemical analyses in the hydroponics assay

#### Measurement of root length and leaf surface area

Root elongation (RE, cm) was determined using ImageJ software (https://www.imagej.nih.gov/ij/download.html), while LSA (cm^2^) was analyzed by UTHSCA (http://ddsdx.uthscsa.edu/dig/itdesc.html) image tool software.

#### Relative water content

RWC was calculated as described by Tounsi et al. (2017), using the formula: RWC (%) = (fresh weight – dry weight)/(saturated fresh weight – dry weight) ×100.

#### Chlorophyll and carotenoid contents

Fresh leaves were weighed, immersed in 80% acetone and kept at 4°C in the dark for 24h. Total chlorophyll and carotenoid contents were measured using a spectrophotometer at 646.6 nm, 663.6 nm, and 450 nm, respectively (de Carvalho et al., 2012; Tounsi et al., 2017).

#### Proline and total soluble sugar contents

Proline content was determined according to Bates et al. (1973). Briefly, fresh leaves (0.25 g) were mixed in 5 mL of 3% (w/v) sulfosalicylic acid and filtered. Then, 2 mL of filtrate were mixed with 2 mL of acidic ninhydrin reagent and 2 mL of glacial acetic acid. The mixture was incubated for 1h at 100°C. After incubation, 4 mL of toluene were added and vigorously mixed for 20 s. The chromophore was aspirated from the aqueous phase and the absorbance measured at 520 nm.

Total soluble sugar (TSS) content was measured in leaves as described by Bouteraa et al. (2022). Fresh leaves (0.2 g) were vigorously mixed in 80% ethanol solution and the mixture was centrifuged for 10 min at 10,000×g at 4°C. One milliliter of supernatant was added to 3 mL of anthrone reagent and the reaction mixture was heated for 10 min at 100°C. After cooling the reaction, optical density was measured at 620 nm.

#### Malondialdehyde and hydrogen peroxide contents

Malondialdehyde (MDA) content, content, a commonly used measure of the extent of lipid peroxidation, was determined according to Draper and Hadley (1990). Fresh leaves were homogenized in 0.1% TCA and centrifuged for 30 min at 12,000×g. One milliliter of supernatant was added to 1 mL of 0.5% TBA, incubated for 30 min at 95°C and then cooled for 10 min. After centrifugation at 12,000×g for 5 min, the absorbance of supernatant was measured at 532 nm and 600 nm.

Hydrogen peroxide (H_2_O_2_) content was measured as described by Velikova et al. (2000). Fresh leaves were homogenized in 0.1% TCA and centrifuged for 30 min at 12,000×g. Thereafter, 0.5 mL of supernatant was mixed with 0.5 mL potassium phosphate buffer (10 mM, pH 7.0) and 1 mL potassium iodide (1 M). The absorbance was determined at 390 nm.

#### Antioxidant activities

Fresh leaves (0.2 g) were homogenized with 2 mL of chilled extraction buffer (50 mM phosphate buffer pH 7.0, 2% PVP, and 1 mM PMSF). The homogenate was centrifuged at 10,000×g for 20 min at 4°C and the supernatant was collected and kept at 4°C to determine the antioxidant activities of the following enzymes: SOD, CAT, POD, and APX. Protein concentration in the different extracts was determined as described by Bradford (1976).

SOD activity was determined by quantifying the enzyme ability to inhibit the photochemical reduction of nitro-blue tetrazolium (NBT) (Djemal and Khoudi, 2016). Briefly, 20 µg of crude enzyme extract was added to the reaction mixture composed of 50 mM phosphate buffer (pH 7.8), 0.1 mM EDTA, 13 mM L-methionine, 2 mM riboflavin, and 75 mM NBT. The mixture was exposed to cool-white, fluorescent light for 15 min and absorbance was determined at 560 nm.

CAT activity was determined by measuring the reduction of H_2_O_2_ at 240 nm (Aebi, 1984). The reaction solution was composed of 50 mM potassium phosphate buffer (pH 7.0), 30 mM H_2_O_2_, and 20 µg of crude enzyme extract.

POD activity was measured as described by Bouteraa et al. (2022) via the guaiacol oxidation method. The reaction mixture was composed of 50 mM phosphate buffer (pH 7), 20 mM guaiacol, 40 mM H_2_O_2_, and 20 µg of crude enzyme extract. The absorbance was determined at 470 nm.

APX activity was determined as described by Nakano and Asada (1981). The reaction mixture contained 50 mM potassium phosphate (pH 7.0), 0.5 mM ascorbate, 0.1 mM H_2_O_2_, and 0.1 mM EDTA in a total volume of 1 ml. The absorbance was measured at 290 nm.

As for non-enzymatic antioxidant molecules, AsA content was determined following the method described by Feki et al. (2017). Briefly, plant material (0.5 g) was vigorously ground in 10 mL of 6% (w/v) TCA solution and then mixed with 2% dinitrophenylhydrazine, followed by addition of one drop of thio-urea. The mixture was boiled for 15 min and cooled for 10 min at room temperature. Thereafter, 5 mL of 80% (v/v) H_2_SO_4_ were added to the mixture at 0°C and the absorbance was measured at 530 nm.

#### Na^+^ and K^+^ contents

Na^+^ and K^+^ concentrations were measured in roots and leaves as previously described by Tounsi et al. (2017). Briefly, tissues were dried at 70˚C and then solubilized in 0.1 N hydrochloric acid for 24h. Each sample was diluted to measure Na^+^ and K^+^ contents using flame spectrophotometry (SpectrAA 220 FS, Varian).

### Cell cycle analysis

To evaluate the effect of salt stress on cell cycle progression, 3-day-old seedlings of 7 DW genotypes, namely, R112+, R112−, R69-9/R5+, R69-9/R5−, R74-10/R112+, R74-10/R112− and cv. OR, and 3 BW genotypes, that is, CS7E(7D), CSTr#12, and CS, were placed in aerated Hoagland solution (Doležel et al., 1999), supplemented with 200 mM NaCl or 0 mM NaCl (control condition), at 22 ± 1°C for 24h. After the salt treatment, roots from 3–6 plants/genotype/treatment were excised and fixed in 2% (v/v) formaldehyde in 1X Tris-HCl buffer pH 7.5 for 10 min at 5°C (Doležel et al., 1992). Root tip nuclei from each plant were isolated in LB01 buffer (Doležel and Lucretti, 1989) by homogenization with a Mini-Turrax T8 with a S10N-5G generator (IKA, Staufen, Germany) for 12 s at 9,500 rpm. After DNA staining with DAPI (4,6-diamidino-2-phenylindole), nuclei were run on a CytoFLEX S flow analyzer (Beckman Coulter Flow Cytometry, Milan, Italy), using as internal reference standard (IRS) 2.5 µm polystyrene microspheres (Alignflow Beads for UV lasers cod. A16502, Thermo Fisher Scientific, Milan, Italy) to monitor instrument stability and to ensure a reliable comparison among experiments. DNA fluorescence emission was evaluated on at least 3,000 DAPI stained nuclei/plant, excited by a violet laser (ext. 405 nm), and the main DAPI fluorescence emission was collected at 525/40 nm. The distribution of cells at each of G1, S and G2 cell cycle phases was investigated by processing data with the cell cycle modeling utility in Kaluza 1.2 software (Beckman Coulter Life Science, IN, USA). The Kaluza tool facilitates determination of the percentage distribution of nuclei in G1, S and G2 phases according to their fluorescence, which is proportional to nuclear DNA content.

### Statistical analysis

For all experiments, datasets of DW and BW genotypes were analyzed separately. Two-way analysis of variance (ANOVA) was performed using the statistical software SPSS V23 (IBM Corp, Armonk, NY, USA) or SYSTAT12 (Systat Software Incorporated, San Jose, CA, USA), where Genotype (G) and Treatment (T) were inserted as main factors. For the second germination assay, Replica (R) was inserted as a covariance, corresponding to the two assay repetitions in time. In all analyses, the second order interaction G × T was also analyzed. When significant F values were observed, a pairwise post-ANOVA analysis was carried out by the Tukey Honestly Significant Difference test (Tukey test) at *p* < 0.05. Principal component analysis (PCA) for each of the DW and BW datasets was performed in R Environment (R Project for Statistical Computing 4.3.3), using functions included in “FactoMineR,” “ggplot2,” and “factoextra” packages.

## Results

The salt (NaCl) stress conditions applied in the present work had an overall major impact on the traits assayed, as shown by several indicators of morpho-physiological, biochemical, and cellular plant features and functions. To the stress, the tested genotypes responded differently, particularly in relation to presence/absence of given *Thinopyrum* introgressions. For the majority of traits described in the following, ANOVA (**Tables 1**, **2**; **Supplementary Table S1**) revealed highly significant differences due to the treatment (T, stressed versus unstressed conditions), to the genotype (G, several introgression versus CLs of DW and BW) and to their interaction (G × T).

**Table 1:** Mean values ± standard error and ANCOVA mean squares of morphological traits after exposure to salt stress of primary (R112+) and secondary (R74-10/R112+) DW recombinant lines (+) and their respective controls, lacking the alien segment (**–**).

Genotype	Trait					
	RL4 (mm)	LL4 (cm)	RL10 (mm)	LL10 (cm)	RDW (g)	LDW (g)
0 mM NaCl						
**R112+**	6.20 ± 0.27 a	2.53 ± 0.11 a	18.16 ± 0.39 ab	12.22 ± 0.24 a	0.05 ± 0.00 ab	0.02 ± 0.00
**R112–**	4.88 ± 0.17 b	2.58 ± 0.10 a	12.45 ± 0.58 c	11.61 ± 0.20 ab	0.04 ± 0.00 b	0.02 ± 0.00
**R74-10/R112+**	5.12 ± 0.18 b	2.17 ± 0.09 b	18.80 ± 0.31 a	10.91 ± 0.22 b	0.06 ± 0.00 a	0.02 ± 0.00
**R74-10/R112–**	5.51 ± 0.20 b	2.13 ± 0.07 b	16.95 ± 0.48 b	10.03 ± 0.20 b	0.05 ± 0.00 ab	0.02 ± 0.00
100 mM NaCl						
**R112+**	3.47 ± 0.13 c	1.62 ± 0.06 c	8.18 ± 0.20 de	8.70 ± 0.20 c	0.03 ± 0.00 c	0.01 ± 0.00
**R112–**	2.50 ± 0.12 d	1.24 ± 0.08 d	5.82 ± 0.24 f	6.69 ± 0.25 d	0.02 ± 0.00 ce	0.01 ± 0.00
**R74-10/R112+**	3.51 ± 0.11 c	1.27 ± 0.05 d	8.99 ± 0.22 d	7.25 ± 0.20 d	0.02 ± 0.00 cd	0.01 ± 0.00
**R74-10/R112–**	3.11 ± 0.13 cd	1.18 ± 0.04 d	7.66 ± 0.13 e	6.90 ± 0.16 d	0.02 ± 0.00 df	0.01 ± 0.00
200 mM NaCl						
**R112+**	0.98 ± 0.07 e	0.50 ± 0.02 e	2.39 ± 0.14 g	1.36 ± 0.13 e	0.01 ± 0.00 f	0.00 ± 0.00
**R112–**	1.16 ± 0.08 e	0.56 ± 0.03 e	2.91 ± 0.13 g	1.26 ± 0.13 e	0.01 ± 0.00 f	0.00 ± 0.00
**R74-10/R112+**	1.32 ± 0.08 e	0.54 ± 0.02 e	3.04 ± 0.13 g	1.17 ± 0.08 e	0.01 ± 0.00 ef	0.00 ± 0.00
**R74-10/R112–**	0.99 ± 0.09 e	0.51 ± 0.02 e	2.13 ± 0.20 g	1.31 ± 0.11 e	0.01 ± 0.00 f	0.00 ± 0.00
ANCOVA						
**G**	11.9***	1.8***	265.7***	44.7***	0.000**	0.000
**T**	907.0***	132.4***	9926.6***	4269.1***	0.020***	0.004***
**G x T**	7.4***	0.9**	95.9***	18.1***	0.000*	0.000
**Rep**	42.5***	2.9**	72.3***	90.1***	0.000	0.000**

RL4, root length 4 days after sowing (DAS); RL10, root length 10 DAS; LL4, leaf length 4 DAS; LL10, leaf length 10 DAS; LDW, leaf dry weight; RDW, root dry weight. LDW and RDW were measured 10 DAS. Letters in each column correspond to ranking of Tukey test at p < 0.05 level for significant Genotype (G) x Treatment (T) interactions; Rep, replica included as a covariant; *, **, and *** indicate significance at p < 0.05, p < 0.01, and p < 0.001, respectively.

**Table 2:** Mean values (%) ± standard error and ANOVA mean squares of nuclei in cell cycle phases G1, S, and G2 of DW (A) and BW (B) introgression and control lines subjected to salt stress versus control conditions.

(A)				
DW lines	G1	S	G2	
0 mM NaCl				
**R112+**	49.11 ± 1.04	17.62 ± 0.47 ab	33.28 ± 1.08 bc	
**R112–**	47.72 ± 0.87	17.82 ± 1.29 ab	33.46 ± 0.78 ac	
**R69-9/R5+**	49.69 ± 0.97	19.10 ± 0.42 a	31.26 ± 1.26 c	
**R69-9/R5–**	49.35 ± 1.62	19.24 ± 2.07 a	31.41 ± 0.56 bc	
**R74-10/R112+**	52.57 ± 1.03	13.44 ± 1.52 ae	33.98 ± 0.83 ac	
**R74-10/R112–**	51.84 ± 1.19	15.49 ± 1.09 ad	32.67 ± 0.44 bc	
**Om Rabia**	47.37 ± 1.05	17.35 ± 0.77 ab	35.27 ± 0.60 ac	
200 mM NaCl				
**R112+**	52.92 ± 1.12	9.65 ± 1.43 e	37.60 ± 2.06 ab	
**R112–**	50.77 ± 1.54	16.63 ± 1.57 ac	32.83 ± 1.17 bc	
**R69-9/R5+**	55.11 ± 0.43	9.97 ± 1.13 de	34.92 ± 1.10 ac	
**R69-9/R5–**	54.86 ± 2.49	12.76 ± 1.15 ae	32.37 ± 1.57 bc	
**R74-10/R112+**	58.28 ± 0.69	12.27 ± 0.68 be	29.46 ± 1.12 c	
**R74-10/R112–**	57.67 ± 1.43	11.67 ± 1.08 ce	30.66 ± 0.96 c	
**Om Rabia**	49.91 ± 2.06	10.53 ± 1.04 de	39.57 ± 1.74 a	
ANOVA				
**G**	61.0***	19.3*	47.2***	
**T**	303.2***	399.5***	11.0	
**G x T**	4.3	21.9**	28.0**	
(B)				
BW lines	G1	S	G2	
0 mM NaCl				
**CS7E(7D)**	55.77 ± 0.68	15.51 ± 0.83	28.73 ± 0.83	
**CSTr#12**	56.25 ± 0.86	15.21 ± 1.06	28.54 ± 0.55	
**CS**	55.19 ± 1.33	16.31 ± 1.37	28.50 ± 1.00	
200 mM NaCl				
**CS7E(7D)**	68.84 ± 1.23	5.19 ± 0.65	25.97 ± 0.72	
**CSTr#12**	67.06 ± 0.89	6.79 ± 0.76	26.14 ± 1.19	
**CS**	68.47 ± 1.23	7.99 ± 0.22	23.55 ± 1.23	
ANOVA				
**G**	1.2	8.6	5.9	
**T**	1184.0***	627.4***	87.9***	
**G x T**	4.8	3.3	4.8	

Letters in columns of S and G2 phases of nuclei of DW genotypes (A) correspond to ranking of Tukey test at p < 0.05 level for genotype (G) x treatment (T) interactions. *, **, and *** indicate significance at p < 0.05, p < 0.01, and p < 0.001, respectively.

### Seed germination

**Figure 3:**
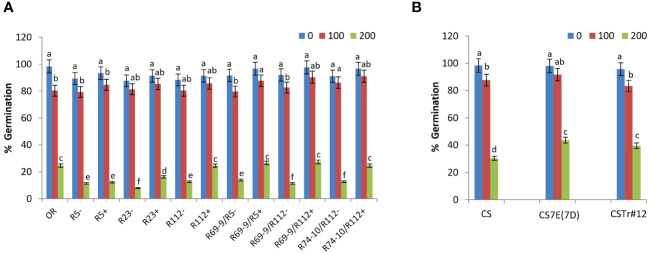
Effect of salt stress application on seed germination. Comparison between DW recombinant (+) versus control (−) lines and cv. OR **(A)** and between BW lines CSTr#12 and CS7E(7D) versus normal CS **(B)**. Values are expressed as means ± SE. Letters above histograms correspond to the ranking of Tukey test at *p* < 0.05 significance level. Color-coded legend: NaCl concentration (mM).

Salt stress application caused a significant reduction in SG ability of all genotypes (**Figure 3**), but with RLs showing, mainly at 200 mM NaCl, superior performance versus their control (“−” sib) lines (CLs). At the highest salt concentration, SG was increased in lines R23+, R112+, R69-9/R5+, R69-9/R112+, R74-10/R112+ versus their controls by 104%, 94%, 90%, 141%, and 94%, respectively (**Figure 3A**). Albeit not significant, at 200 mM NaCl recombinants R69-9/R5+ and R69-9/R112+ exhibited even higher SG (+8% and +10%, respectively) than the salt-tolerant Tunisian cv. OR. Similarly in the BW lines, although to a lesser extent than in DW, SG was significantly improved by the presence of the entire 7E chromosome in CS7E(7D) and most of the 7el_1_ in CSTr#12 (+43% and +30%, respectively) into the background of the recipient BW cv. CS (**Figure 3B**).

### Plant growth and cell cycle progression

**Figure 4:**
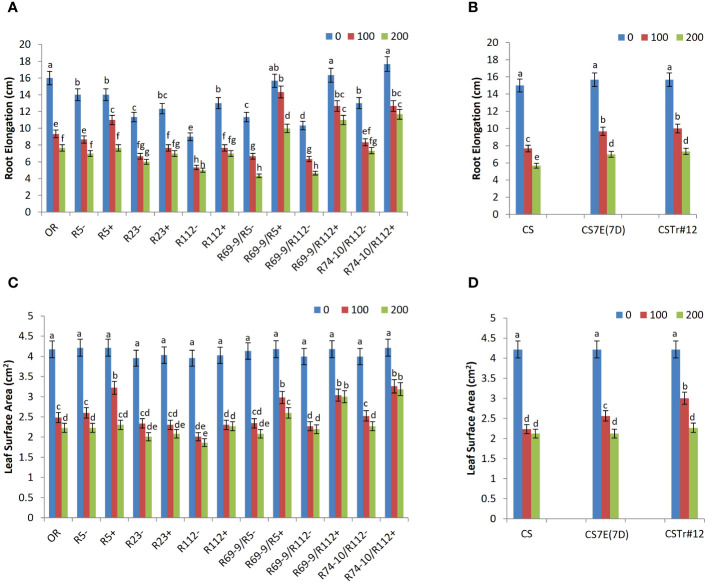
Effect of salt stress application on root elongation **(A, B)** and leaf surface area **(C, D)** of wheat lines. Comparison between DW recombinant (+) versus control (−) lines and cv. OR **(A, C)** and between BW lines CSTr#12 and CS7E(7D) versus normal CS **(B, D)**. Values are expressed as means ± SE. Letters above histograms correspond to the ranking of Tukey test at *p* < 0.05 significance level. Color-coded legend: NaCl concentration (mM).

In the of hydroponic assay, already under control conditions significant differences for RE were displayed by primary RL R112+ and all secondary RLs (R69-9/R5+, R69-9/R112+, and R74-10/R112+) versus their CLs. At 100 mM NaCl compared to 0 mM, the smallest decline of RE was observed in R5+ and in all the secondary RLs, among which R69-9/R5+ showed no significant stress effect on this trait. The remaining genotypes, including cv. OR, had conspicuous reductions of RE already at this salt concentration (**Figure 4A**). Then, at 200 mM NaCl, secondary RLs R69-9/R5+, R69-9/R112+, and R74-10/R112+ not only outperformed their “−” controls (+130%, +135%, +59%, respectively) and OR (+30%, +43%, +52%, respectively) but also their corresponding primary types, R5+ and R112+ (**Figure 4A**). Some incremental effect on RE was also observed at both NaCl concentrations in BW introgression lines, CS7E(7D) and CSTr#12, with respect to normal CS (**Figure 4B**), but neither one reached the high values of DW secondary RLs.

Likewise, under high salt stress, LSA was considerably larger in R69-9/R5+, R69-9/R112+, and R74-10/R112+ than in their CLs (+24%, +36%, and +40%, respectively) and in OR (+16%, +34%, and +42%, respectively, **Figure 4C**). Among primary RLs, R5+ did not differ from secondary types at 100 mM NaCl, but at 200 mM it did not maintain the same LSA values as those of secondary types. As for RE, also LSA values were higher in DW secondary RLs than in CS7E(7D) and CSTr#12, even at 200 mM NaCl (**Figure 4D**), supporting the hypothesis that presence of both alien segments (7el_1_L + 7EL) has an additive effect on both growth traits compared with their separate condition.

As for plants grown in Petri dishes and directly exposed to salt stress from germination up to 10 DAS, the statistical analysis showed significant differences for most of the measured parameters, namely, RL4, RL10, LL4, and LL10 and LDW and RDW at 0 mM and 100 mM NaCl (**Table 1**). The presence of the 7el_1_ *Th. ponticum* segment in R112+ was associated with a greater root length when compared to R112− at 0 mM NaCl (+27%) and at 100 mM NaCl (+39%), though not at higher salt concentration. On the other hand, at 200 mM NaCl, in the frame of a general major impairment of root growth, presence of the composite 7el_1_ + 7E segment in R74-10/R112+ led to increased RL4 by 33% and RL10 by 42% versus its absence (R74-10/R112−). Leaf growth was enhanced in R112+ versus R112− (+30% for LL4 and LL10), but only under the milder salt stress condition (100 mM), and a similar result was observed also for root biomass (+50% for RDW). No significant difference was observed in R74-10/R112+ versus R74-10/R112− for LL4, LL10, LDW, and RDW, at all salt concentrations (**Table 1**).

Since at cellular level plant growth depends on cell proliferation through the mitotic cycle and subsequent cell expansion, we investigated whether salt treatment differentially affected cell cycle progression in a subset of DW RLs (R112+, R69-9/R5+, and R74-10/R112+), their CLs and cv. OR, as well as in BW lines CS7E(7D), CSTr#12, and the CS control. DAPI-stained nuclei isolated from root tips of young seedlings exposed to 0 or 200 mM NaCl were analyzed by flow cytometry. Based on fluorescence emission, the percentage distribution of nuclei in G1, S and G2 phases was determined (**Table 2**). As for DW lines, in all of them salt stress versus the unstressed condition caused an increase of nuclei in G1, ranging from 5.4% in cv. OR to around 11% in R69-9/R5 and R74-10/R112, with no major difference between each RL and its CL. A similar variation was also detected for nuclei in G2 of stressed versus unstressed RLs R112+ and R69-9/R5+ and cv. OR (+13%, +11.7%, and +12.2%, respectively), which was not exhibited by R112– and R69-9/R5–. Conversely, R74-10/R112+ and, to a minor extent, its R74-10/R112– CL, showed a reduction of cell population in G2 (–13.3% and –6.1%, respectively) in stressed versus unstressed plants. On the other hand, a more prominent and unidirectional genotype effect upon salt treatment was displayed by S phase data, with all genotypes exhibiting a reduction of nuclei in this phase at the 200 mM versus 0 mM NaCl (**Table 2**). The decrease was particularly strong in R112+ (–45%) compared with R112– (–12%) (**Table 2A**; **Supplementary Figure S1**), the difference between the two lines resulting in the only significant one among the RL versus CL pairs subjected to this analysis. As the two lines showed almost identical figures of nuclei in S phase under the unstressed condition (**Table 2A**; **Supplementary Figure S1**), the difference under stress is clearly ascribable to the *Th. ponticum* segment present in R112+. The same comparison showed a less dramatic difference between R69-9/R5+ and R69-9/R5– (–48% and –34% at 200 mM NaCl versus 0 mM NaCl, respectively), and between R74-10/R112+ versus its CL (–9% and –25%, respectively). The Tunisian cv. OR had a 40% reduction at 200 mM salt. Regarding the BW lines, ANOVA revealed no significant genotype effect for all cycle phases (**Table 2B**). Both introgression lines and their CS control showed a similar increment of percentage nuclei in G1 (from 19% to 24%), a decrease in G2 [by 9.6%, 8.4% and 17% in CS7E(7D), CSTr#12 and CS, respectively], and a pronounced reduction in S phase (by 55%, 66%, and 51% for the same genotypes as above). Thus, presence/absence of either *Thinopyrum* introgression did not seem to have in BW a significant impact on cell cycle modulation due to salt stress, at least under the experimental conditions applied here.

### Relative water content

**Figure 5:**
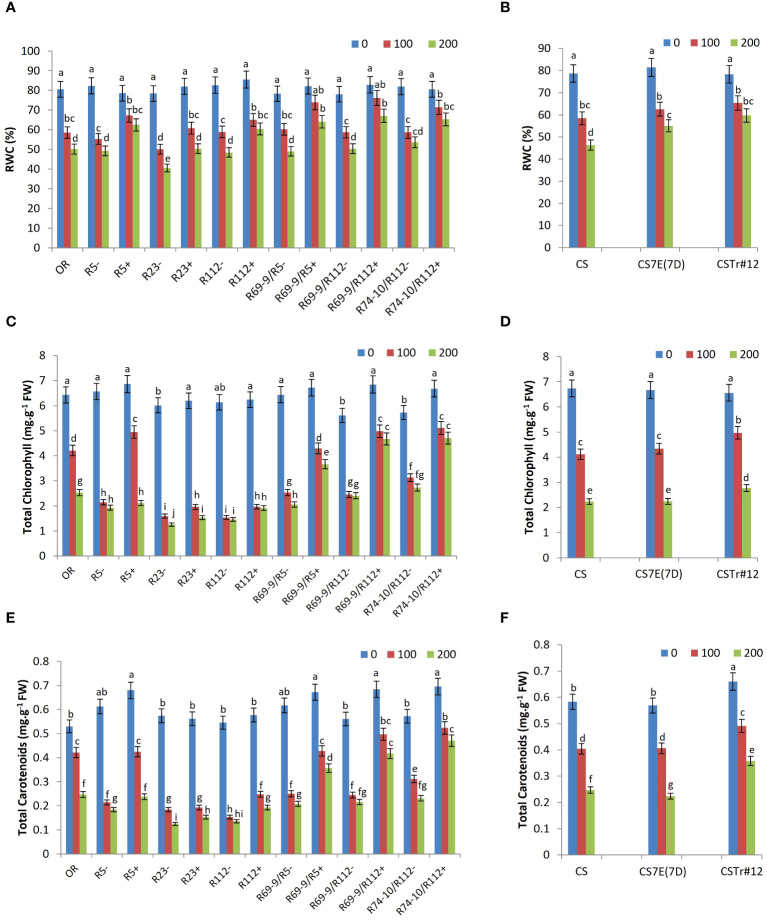
Relative water content (RWC, **A, B**), total chlorophyll **(C, D)**, and carotenoids **(E, F)** of wheat lines subjected to salt stress application. Comparison between DW recombinant (+) versus control (−) lines and cv. OR **(A, C, E)** and between BW lines CSTr#12 and CS7E(7D) versus normal CS **(B, D, F)**. Values are expressed as means ± SE. Letters above histograms correspond to the ranking of Tukey test at *p* < 0.05 significance level. Color-coded legend: NaCl concentration (mM).

The ability to maintain high RWC is a critical strategy to mitigate the negative effect of salinity. All DW-*Thinopyrum* spp. RLs+ displayed a better water uptake ability compared with CLs at both 100 mM NaCl and 200 mM NaCl. At 100 mM NaCl, the RWC increment spanned from a minimum of 10% (R112+) to a maximum of 29% (R69-9/R112+), the difference being significant for R5+, R23+, R69-9/R112+, and R74-10/R112+. At 200 mM NaCl, the difference increased, being on average nearly 27% higher in RLs+ versus their “−” controls, with the greatest increments exhibited by the secondary recombinants R69-9/R5+ and R69-9/R112+ (+30% and +33%, respectively; **Figure 5A**). Notably, R69-9/R5+, R69-9/R112+, and R74-10/R112+ showed the highest RWC absolute values under both saline conditions, better than those of primary types and of the Tunisian cv. OR. For this trait, no significant difference was observed in BW introgression lines compared with normal CS at 100 mM salt, while a higher water content was retained in leaves of CS7E(7D) substitution line and CSTr#12 at 200 mM, although absolute values were inferior to those of DW RLs, particularly secondary types with “nested” *Th. ponticum + Th. elongatum* introgressions (**Figures 5B** versus **5A**).

### Photosynthetic pigments

All DW-*Thinopyrum* spp. recombinant genotypes showed significantly higher total chlorophyll and total carotenoid content in comparison with their CLs under salt stress conditions (**Figures 5C, D**). With respect to the untreated condition, the genotypes that best maintained their photosynthetic pigments were R69-9/R112+ and R74-10/R112+, showing similar values under both NaCl concentrations, slightly higher than those of R69-9/R5+, but remarkably higher than those of primary recombinants (R5+, R112+, and R23+) and cv. OR, particularly at 200 mM NaCl. At this concentration, total chlorophyll of R69-9/R5+, R69-9/R112+, and R74-10/R112+ exceeded that of CLs by 78%, 94%, and 72%, respectively, and that of cv. OR by 44%, 84%, and +86%, respectively. Moreover, comparing secondary recombinants versus corresponding primary types, the better performance of the former ones clearly emerged: R69-9/R5+ exceeded R5+ by 73% and so was for R69-9/R112+ and R74-10/R112+ versus R112+ (+143% and +145%, respectively, **Figure 5C**).

A similar picture was true for total carotenoid content, considerably higher in the three secondary DW RLs versus their controls (+71%, +93%, and +103%, respectively) and cv. OR (+44%, +68%, and +90%, respectively, **Figures 5E, F**). Under 200 mM NaCl, a significant difference was also observed in R69-9/R5+ versus R5+ (+50%) and in R69-9/R112+ and R74-10/R112+ versus R112+ (+116% and +144%, respectively), which confirmed the more relevant contribution of the “nested” introgression than the sole *Th. ponticum* introgression to these parameters. On the other hand, at the hexaploid level, presence of the entire 7E chromosome from *Th. elongatum*, as in the CS7E(7D) substitution line, was not apparently beneficial for all photosynthetic pigment content, whereas some advantage versus normal CS seemed to be associated with the *Th. ponticum* introgression of CSTr#12 (**Figures 5D, F**).

### Osmolyte accumulation

**Figure 6:**
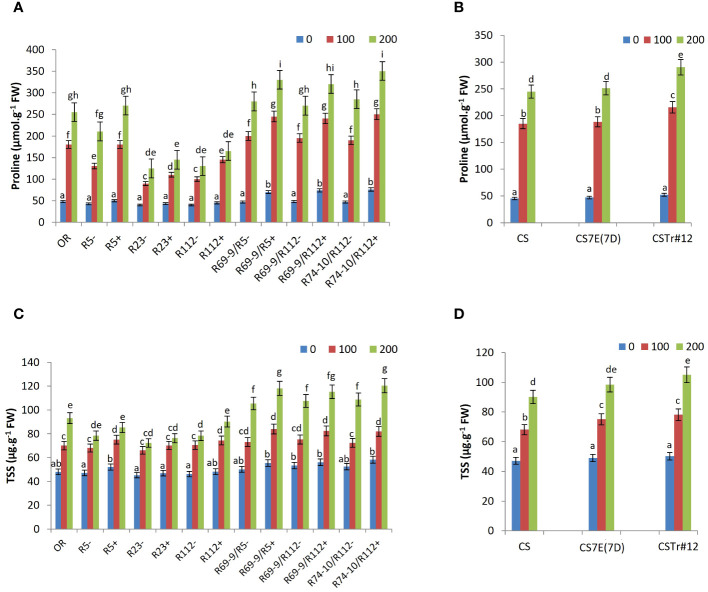
Effect of salt stress application on proline **(A, B)** and total soluble sugars (TSS; **C, D**). Comparison between DW recombinant (+) versus control (−) lines and cv. OR **(A, C)** and between BW lines CSTr#12 and CS7E(7D) versus normal CS **(B, D)**. Values are expressed as means ± SE. Letters above histograms correspond to the ranking of Tukey test at *p* < 0.05 significance level. Color-coded legend: NaCl concentration (mM).

A significant accumulation of proline and TSS was observed in all wheat genotypes under salt stress versus control conditions, significantly increasing from the mild stress of 100 mM to 200 mM NaCl (**Figure 6**). Among DW RLs, secondary RLs R69-9/R5+, R69-9/R112+, and R74-10/R112+ reached the highest values, which were significantly higher than those of their CLs under both salt concentrations (**Figure 6A**). Compared to secondary RLs, lower proline amounts were displayed by cv. OR and the R5+ RL (−30% to 40% at 100 mM and −25% at 200 mM). Rather unexpectedly, primary RLs R112+ and R23+, and so their respective CLs, produced the lowest proline levels, with small incremental effects of their *Th. ponticum* segments, albeit significant at 100 mM (**Figure 6A**). As to the BW lines, proline content was not affected by *Th. elongatum* chromosome 7E substitution in place of wheat 7D, whereas presence of the *Th. ponticum* introgression (CSTr#12) onto chromosome 7A caused a 20% increase in comparison with normal CS at both salt concentrations (**Figure 6B**).

An analogous trend was observed for TSS levels, which were slightly higher in both BW introgression lines versus CS at 100 mM salt but accumulated in significantly higher amount in CSTr#12 only at 200 mM (**Figure 6D**). Also, for the DW lines the tendency for TSS accumulation was comparable to that of proline, with a clearer discrimination between primary and secondary RLs. The latter, particularly under the most stressful treatment (200 mM NaCl), had the highest values in absolute terms, in most cases significantly differing from their CLs and in all cases significantly exceeding the primary RLs and cv. OR (**Figure 6C**). For TSS, the behavior of primary RLs was more uniform across lines, with slightly lower values of R23+ versus R112+ and R5+.

### Biochemical analyses of oxidative stress

At the cellular level, excessive salt induces oxidative stress, due to overproduction and accumulation of harmful ROS, as well as membrane damage. To further elucidate the response of DW RLs to salt application, the MDA and hydrogen peroxide (H_2_O_2_) content as well as the activities of SOD, CAT, POD, and APX enzymes were determined.

### MDA and H_2_O_2_ content

**Figure 7:**
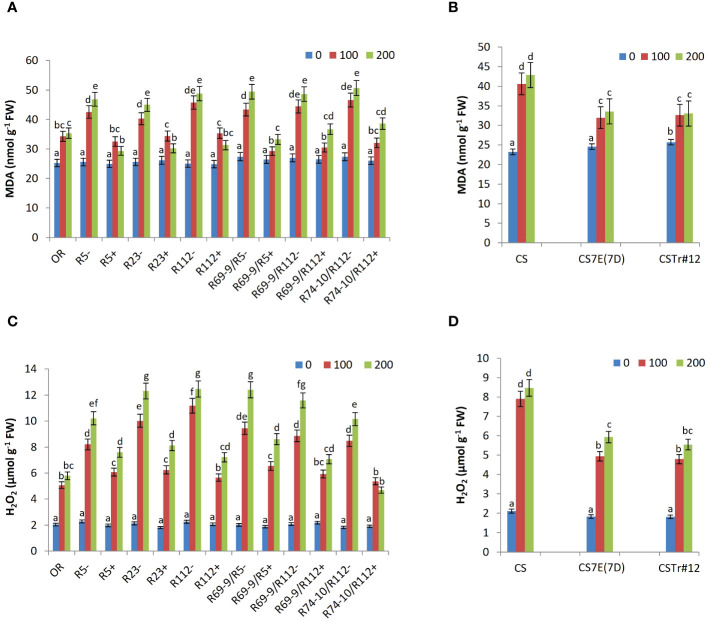
Malondialdehyde (MDA; **A, B**) and hydrogen peroxide (H_2_O_2_) content **(C, D)** in leaf tissues of wheat lines following salt stress exposure. Comparison between DW recombinant (+) versus control (−) lines and cv. OR **(A, C)** and between BW lines CSTr#12 and CS7E(7D) versus normal CS **(B, D)**. Values are expressed as means ± SE. Letters above histograms correspond to the ranking of Tukey test at *p* < 0.05 significance level. Color-coded legend: NaCl concentration (mM).

Under control conditions, MDA and H_2_O_2_ contents were similar in all wheat genotypes (**Figure 7**). However, under salt stress conditions, the accumulation of MDA and H_2_O_2_, both indicative of higher stress impact, was significantly lower in DW and BW lines carrying *Thinopyrum* spp. introgressions with respect to their CLs lacking any alien transfer and in cv. OR. Under 200 mM NaCl, the MDA content of R5+, R23+, R112+, R69-9/R5+, R69-9/R112+, and R74-10/R112+ was significantly reduced by 37%, 32%, 35%, 32%, 24%, and 23% compared with their CLs, respectively (**Figure 7A**). Likewise, these six lines showed a significant reduction in H_2_O_2_ accumulation (−25%, −34%, −42%, −30%, −38%, and −53% versus their respective CLs; **Figure 7C**). For both MDA and H_2_O_2_, primary and secondary recombinants had a largely similar behavior, which suggests a common genetic control, possibly at the level of the shared *Th. ponticum* segment (see **Figure 1**). However, it is worth noting that R74-10/R112+ exhibited the lowest H_2_O_2_ levels, particularly at 200 mM NaCl, even compared to OR, which showed lower amounts of H_2_O_2_ than the other DW RLs (**Figure 7C**). Regarding the BW lines, the effect of their *Th. ponticum* or *Th. elongatum* introgressions had a comparable effect on reducing both MDA and H_2_O_2_ accumulation as compared with the CS performance (**Figures 7B, D**).

### Antioxidant activities

The ability of plants to neutralize ROS and limit their harmful effects is due to the presence of efficient scavenging systems, involving enzymatic and non-enzymatic antioxidants, including SOD, CAT, POD, and APX enzymes, as well as ascorbate.

**Figure 8:**
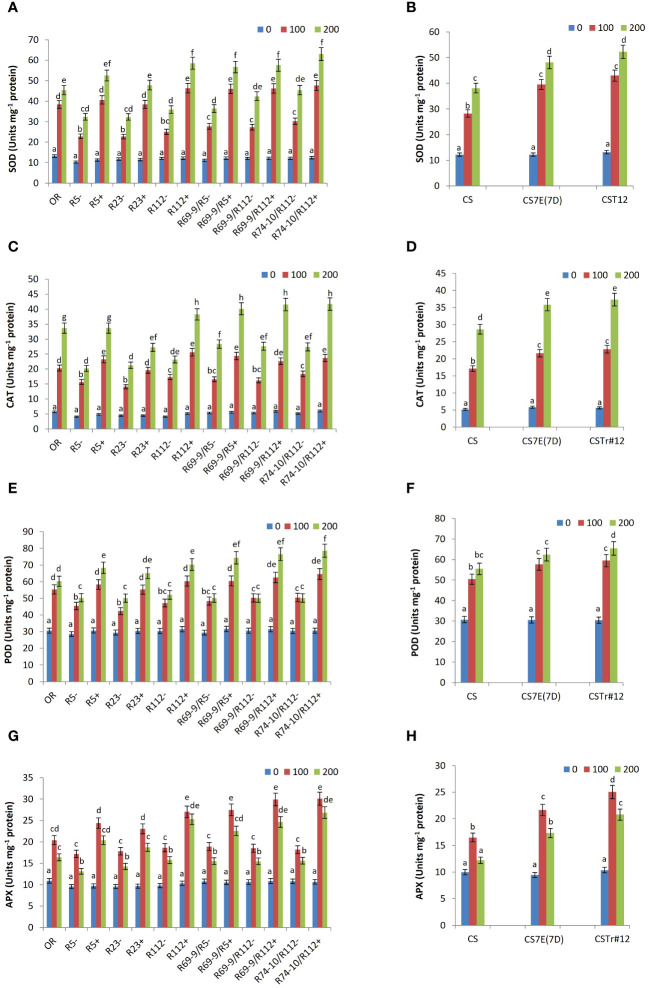
Effect of salt stress application on the activity of the following antioxidant enzymes: superoxide dismutase, SOD **(A, B)**, catalase, CAT **(C, D)**, peroxidase, POD **(E, F)** and ascorbate peroxidase, APX **(G, H)**. For each enzyme, DW recombinant lines (+) are compared to the respective control lines (−) and cv. OR **(A, C, E, G)** and BW lines CSTr#12 and CS7E(7D) to normal CS **(B, D, F, H)**. Values are expressed as means ± SE. Letters above histograms correspond to the ranking of Tukey test at *p* < 0.05 significance level. Color-coded legend: NaCl concentration (mM).

SOD, which catalyzes the conversion of superoxide radical (O_2_^−^) to hydrogen peroxide (H_2_O_2_), is the first enzyme to be involved in ROS removal. Whereas under control (0 mM salt) condition its activity was similar among DW lines, it significantly increased in all RLs compared to CLs lacking the alien segments, in addition that in OR (**Figure 8**). Maximum values were reached under the high salt stress condition of 200 mM, when SOD activity of R5+, R23+, R112+, R69-9/R5+, R69-9/R112+, and R74-10/R112+ exceeded that of respective controls by 62%, 48%, 62%, 55%, 35%, and 38%, respectively. In recombinant genotypes R112+, R69-9/R5+, R69-9/R112+, and R74-10/R112+ SOD activity reached the highest values, resulting significantly higher than those of the Tunisian salt tolerant cv. OR (**Figure 8A**). BW introgression lines also showed a significantly augmented SOD activity versus normal CS in both NaCl concentrations, with no statistical difference between introgression types (**Figure 8B**).

Likewise, salt stress caused in all wheat RLs a significant increase in CAT activity, reaching the highest values in plants treated with 200 mM NaCl. Under this condition, the maximum increase was observed in R5+, R112+, R69-9/R5+, R69-9/R112+, and R74-10/R112+ (+66%, +65%, +41%, +50%, +52%, respectively versus their respective controls), with enzyme peaks exhibited by secondary RLs (**Figure 8C**). Notably, in the latter recombinant types, CAT activity was significantly enhanced by about 20% versus cv. OR (**Figure 8C**). BW introgressions behaved similarly as described for SOD (**Figure 8D**). Similarly, POD activity was significantly higher in DW RLs versus CLs at both NaCl concentrations, the increment varying from 30% to 56% under high salt stress (200 mM NaCl) (**Figure 8E**). As for the previous enzymes, at this concentration secondary RLs displayed the highest values of POD activity with respect to the primary types and to cv. OR. On the other hand, at the hexaploid level, both introgression lines also increased POD activity under salt stress compared with normal CS, with no major difference at the two NaCl concentrations, except for CSTr#12 (*Th. ponticum* introgression), which had a more intense activity at 200 mM compared with the *Th. elongatum* substitution line CS7E(7D) (**Figure 8F**).

**Figure 9:**
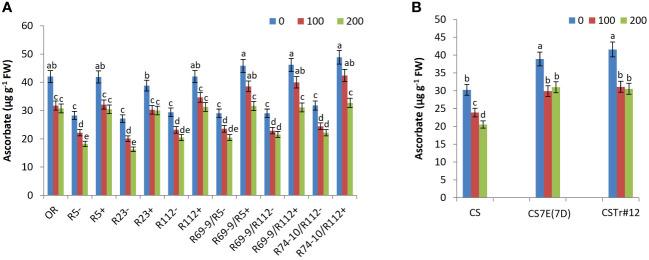
Ascorbate content in DW recombinant lines (+), the respective control lines (−) and in cv. OR **(A)** as well as in BW lines CSTr#12 and CS7E(7D) lines and normal CS **(B)**. Values are expressed as means ± SE. Letters above histograms correspond to the ranking of Tukey test at *p* < 0.05 significance level. Color-coded legend: NaCl concentration (mM).

As for activity of APX, a highly effective enzyme in H_2_O_2_ scavenging, a considerable increase was recorded in all wheat-*Thinopyrum* spp. lines under salt stress treatments compared with control (untreated) conditions, with presence of any *Thinopyrum* introgression (at both ploidy levels) corresponding to a significantly higher activity than that exerted by the respective CLs (**Figures 8G, H**). Interestingly, however, after exposure to the 100 mM salt treatment, all lines reached their peak APX activity, which then somewhat declined at 200 mM salt, although to a variable extent, sometimes significantly, in others not so (**Figures 8G, H**). This trend matches with that of ascorbate (see ahead, **Figure 9**), the specific electron donor used by APX to reduce H_2_O_2_ to H_2_O (e.g., Caverzan et al., 2012; Pandey et al., 2017), whose availability may thus represent the limiting factor of this reaction of the AsA-GSH cycle. In all cases, among DW lines those that exhibited the highest values (not significantly different at 100 and 200 mM NaCl) were RLs R112+ and its derivatives R69-9/R112+ and R74-10/R112+, while lower APX activity was detected in the remaining RLs and in OR (**Figure 8G**). Similarly to the DW evidence, indicating a promoting activity within the R112 segment shared by the mentioned three lines, the BW 7el_1_ CSTr#12 introgression line to be able to induce a higher APX activity than CS7E(7D) substitution line versus the CS control (**Figure 8H**).

Given the many direct and indirect protective functions against oxidative stress brought about by ascorbate (e.g., Akram et al., 2017; Hasanuzzaman et al., 2019), its content is expected to undergo some decline after exposure to stress (see, e.g., Feki et al., 2017). However, the reduction versus the untreated condition was significantly lower in all DW and BW lines possessing *Thinopyrum* spp. introgressions at both 100 and 200 mM NaCl (**Figure 9**). After the more extreme treatment, ascorbate content of DW RLs R5+, R23+, R112+, R69-9/R5+, R69-9/R112+, and R74-10/R112+ reached similar values to each other and to cv. OR, exceeding that of respective CLs by 67%, 84%, 35%, 54%, 44%, and 47%, respectively (**Figure 9A**). However, RLs with *Th. ponticum* + *Th. elongatum* segments, namely, R69-9/R5+, R69-9/R112+, and R74-10/R112+, not only displayed the highest values in controlled conditions but also a non-significant reduction at 100 mM NaCl (**Figure 9A**). In BW introgression lines, presence of either chromosome 7E or 7el_1_ (the latter in CSTr#12) conferred higher ascorbate amount to recipient CS in control condition and a minor and comparable reduction after salt exposure, irrespective of salt concentration (**Figure 9B**).

### Na^+^ and K^+^ contents in roots and leaves

**Figure 10:**
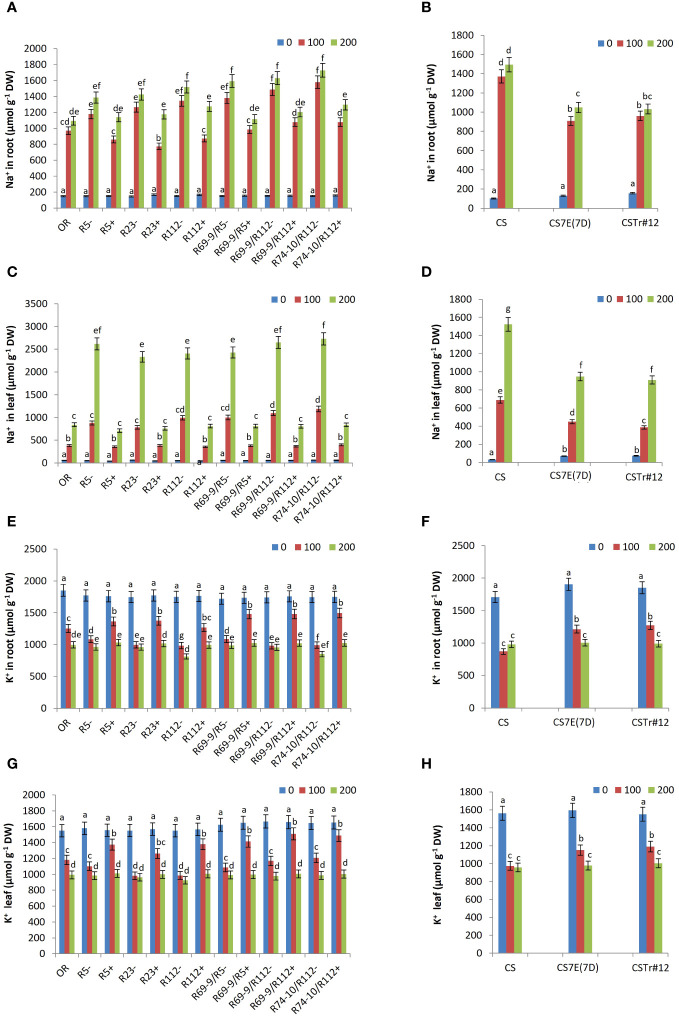
Effect of salt stress treatment on Na^+^ and K^+^ content in roots and leaves of DW-*Thinopyrum* spp. recombinant (+) and corresponding control (−) lines and in cv. OR **(A, C, E, G)**, as well as in BW introgression lines CSTr#12 and CS7E(7D) and in normal CS **(B, D, F, H)**. Values are expressed as means ± SE. Letters above histograms correspond to the ranking of Tukey test at *p* < 0.05 significance level. Color-coded legend: NaCl concentration (mM).

Controlling sodium homeostasis is considered as a key determinant of salt stress tolerance. Thus, Na^+^ and K^+^ ion concentration was measured in roots and leaves under control and salt stress conditions. Irrespective of genotype, the dynamics of Na^+^ accumulation differed in the two organs. In roots, directly exposed to the saline solution, Na^+^ reached in most lines the highest values already after 100 mM salt exposure, with minor increments at 200 mM, while in leaves the increment in salt concentration in the medium determined a considerable Na^+^ surge, particularly in the more sensitive lines (**Figure 10**). However, in both organs of all wheat-*Thinopyrum* spp. lines, a substantial reduction in Na^+^ content versus CLs was observed both under control conditions (constitutive) and after salt exposure (**Figures 10A–D**). All stress-induced differences were significant at 100 mM, and still were at 200 mM NaCl in roots of DW RLs R112+, R69-9/R5+, R69-9/R112+, and R74-10/R112+ (–16%, –30%, –26%, and –25% versus. CLs, **Figure 10A**). The trend was largely similar in both BW introgression lines versus CS (**Figure 10B**). The difference between genotypes carrying or lacking *Thinopyrum* spp. introgressions became even more evident when leaves were considered, with around 70% lower Na^+^ values in DW RLs (and so cv. OR) versus CLs (**Figure 10C**), suggesting that the presence of alien segments efficiently contributes to controlling the amount of sodium delivered from roots to leaves. Likewise, CS7E(7D) and CSTr#12 had much lower Na^+^ concentration in their leaves, both in untreated condition and under salt stress compared with CS (**Figure 10D**).

On the other hand, no significant difference was observed in K^+^ content between DW and BW lines, with or without *Thinopyrum* introgressions, under unstressed conditions (**Figures 10E–H**). However, under 100 mM NaCl, a higher K^+^ ion concentration was observed in roots and leaves of all wheat-*Thinopyrum* spp. lines compared with “wheat-only” controls. Under this condition, the increase of K^+^ content in roots ranged from 26-38% in R5+, R23+, R112+ and R69-9/R5+, to 50%–60% in R69-9/R112+ and R74-10/R112+ (**Figure 10E**), and from 23 to about 40% in their leaves (**Figure 10G**). In cv. OR, K^+^ content was lower than that of DW-*Thinopyrum* spp. RLs, remaining more similar to that of CLs, especially in leaves (**Figures 10E, G**). Exposure to 100 mM NaCl similarly led to a significantly increased K^+^ concentration in roots and leaves of CS7E(7D) and CSTr#12 in comparison with cv. CS (**Figures 10F, H**), although the increase was of minor extent with respect to that observed at the DW level. Under 200 mM NaCl, root and leaf K^+^ content decreased in all lines (except for CS) with respect to the 100 mM condition, more markedly in DW lines, reaching comparable levels across genotypes (**Figures 10E–H**).

### Principal component analysis

**Figure 11:**
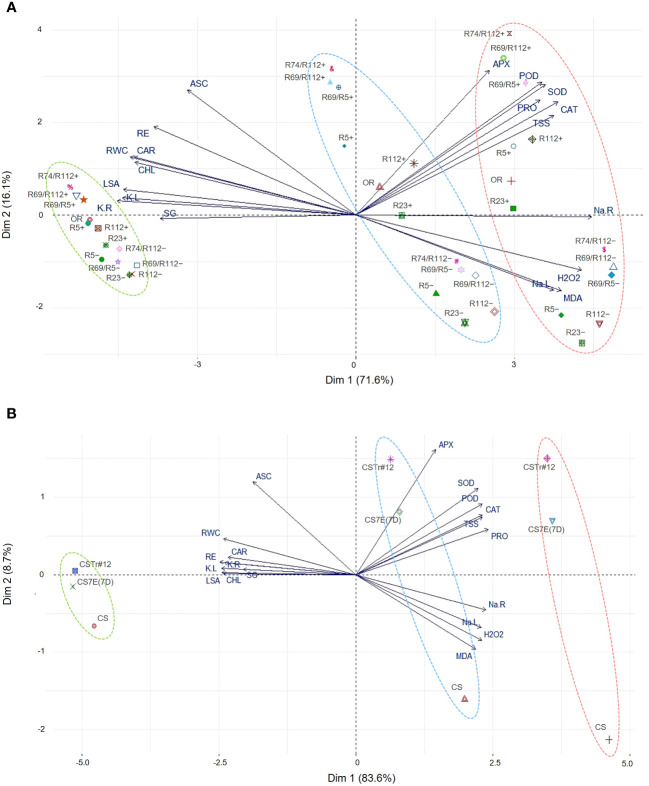
Principal component analysis (PCA) for morpho-physiological and biochemical traits assessed on DW **(A)** and BW **(B)** genotypes grown in hydroponics under salt stress (100 mM NaCl and 200 mM NaCl) and control (0 mM NaCl) conditions. Some DW recombinant (+) and control (−) lines were indicated by shortened names, with R69/R5, R69/R112, and R74/R112 standing for R69-9/R5, R69-9/R112, and R74-10/R112, respectively. For vector-associated trait acronyms/abbreviations, see the list of abbreviations. Color-coded ellipses identify different NaCl treatments: 0 mM, green; 100 mM, blue; 200 mM, red.

To identify traits that are majorly responsible for the difference between genotypes in the response to salt stress, PCAs were performed on the 19 traits measured in both DW and BW groups of materials grown in hydroponics (**Figure 11**). The first two principal components (PCs) explained 87.7% and 92.3% of the variation for DW and BW genotypes, respectively. The impact of the analyzed traits on the observed variability was stronger for DW than BW genotypes, as shown by the bigger distance of the trait vectors form the origin of biplots in the former group. Overall, for both genotype groups the traits clustered in a similar way, indicating the existence of a similar response mechanism in the two species, determined by the donor introgressions in BW lines and their chromosomally engineered smaller fractions in DW lines. PC1 clearly separated the genotype behavior under the control condition (0 mM) from that under salt stress (100 mM and 200 mM). Two trait clusters largely contributed to this separation and were positively correlated with PC1 (**Figures 11A, B**): the first included the traits associated with the observed burst in antioxidant enzymatic activity (APX, POD, SOD, and CAT) and accumulation of osmoprotectants (PRO and TSS), while the other included traits associated with Na^+^ content in leaves and roots (Na.L and Na.R), membrane stability (MDA) and oxidative stress (H_2_O_2_). PC2 separation clearly discriminated introgression carrier (+) and non-carrier (−) lines under stress, being positively correlated with the group of APX, POD, SOD, CAT, PRO, TSS, and ASC traits. In the case of DW lines, PC2 was also positively correlated with root growth (RE) and leaf physiological traits (RWC, CHL and CAR, **Figure 11A**). The PC2 showed limited contribution to the variation of K^+^ content (K.R, K.L), leaf size (LSA) and germination rate (SG) for both DW and BW lines. This was also true for Na^+^ content in roots (Na.R) for DWs, indicating its lower impact on the response to salt stress by these genotypes.

## Discussion

Besides their direct use in saline environments for the many beneficial impacts they can provide to land and users (Flowers and Colmer, 2015; Sheikh-Mohamadi et al., 2022; Tong et al., 2022; Li et al., 2023), wheatgrasses (*Thinopyrum* spp.) have been the target of several attempts to transfer into cultivated *Triticum* spp. at least the major genetic determinants of their halophytic behavior. In fact, many hybrids, partial or complete amphiploids, and even some segmental introgressions have been obtained, but very few of them, if any, have been advanced to the variety status, ready to reach the farmers field (e.g., Colmer et al., 2006; Farooq, 2009; Mullan et al., 2009; Yuan and Tomita, 2015; Mujeeb-Kazi et al., 2019). In the face of rampant global salinity, one reason for this can be identified in the inherently complex nature of plant salinity tolerance, which, as with other abiotic stresses, has a typically polygenic control, where both dominance and additive effects are important for inheritance of the many contributing traits. This was shown to be the case also for the diploid *Th. elongatum*, whose genome partitioning into individual chromosomal components added to or substituted into BW cv. CS confirmed interactive effects to underlie the tolerant phenotype conferred by the entire genome (as in the CS-*Th. elongatum* amphiploid), but also highlighted the existence of major, dominant contributions independently associated to certain chromosomes, including 7E (see Introduction).

These early observations represented an important prerequisite knowledge and support to the research described here, based on a finely tuned chromosome engineering strategy which, while targeting short-sized transfers to optimize interspecific compensation and minimize linkage drag, also aimed at alien gene pyramiding by “nesting” via homoeologous recombination small chromosomal segments from closely related *Thinopyrum* species (Ceoloni et al., 2017; Kuzmanović et al., 2019). As a result, within the group 7 chromosome portions that were transferred from *Th. ponticum* (7el_1_L arm) and *Th. elongatum* (7EL arm) into the primary (7el_1_L) and secondary (7EL nested into 7el_1_L) wheat RLs, several disease resistance genes, quality and yield-contributing traits could be allocated, thanks to the various chromatin breakpoints characterizing each recombinant chromosome (see **Figure 1** and Kuzmanović et al., 2014, 2021). Thus, stimulated by consolidated literature information and by our previous extensive work on the same durum (DW) wheat near-isogenic RLs employed here (see, e.g., Ceoloni et al., 2015; Kuzmanović et al., 2018, 2019, 2021; Fanelli et al., 2023; Giovenali et al., 2023), we have exposed such RLs, along with the original BW-*Thinopyrum* spp. donor lines, to moderate (100 mM) and high (200 mM) salt (NaCl) stress, to verify whether their 7el_1_L and 7EL specific portions could also contribute to salinity tolerance.

### The impact of salt treatment on morpho-physiological, cellular, and biochemical features

A highly positive stress response by all wheat-*Thinopyrum* spp. introgressions when compared with their CLs lacking any alien portion was evident from the early experimental phases, starting with SG. At 200 mM NaCl concentration, when SG ability was significantly affected in all genotypes, no difference was observed between DW RL R5+ and its R5– control, whereas a differential behavior was exhibited by primary RLs with larger 7el_1_L segments (R112+ and R23+, **Figure 1**) and by all secondary RLs versus their respective CLs. Thus, not only an SG promoting factor is likely to reside within the 7el_1_L chromatin proximal to the R5+ breakpoint (**Figure 1**), but given the positive performance of R69-9/R5+ (in which this 7el_1_L stretch is absent, see **Figure 1**), one can also hypothesize that another incremental factor might be located within the most distal 7EL segment, apparently working in a non-additive manner. This reasoning seems to be supported by the results of BW lines, in which both the presence of the complete 7E and of most of 7el_1_ led to a similar increment (**Figure 3**). In all cases, enhancement of SG is a highly positive attribute, as this is the most critical period in the life cycle of salt-affected plants, a period that is extremely sensitive to high salinity and crucial for seedling survival and growth (Yang et al., 2014). Whether caused by osmotic stress that hinders water uptake and/or by ionic toxicity, salinity inhibits cell division and expansion (see ahead), and so the activity of some key enzymes, ultimately reducing utilization of seed reserves (El-Hendawy et al., 2019). In this view, the use of *Thinopyrum* introgression lines appears as a valuable strategy to respond to the need of boosting cereal SG in saline environments (El Sabagh et al., 2021; Rossini et al., 2024).

Traits indicating seedling growth ability under salt stress, i.e. RE and LSA, clearly showed the superior performance of DW secondary RLs not only versus their CLs but also versus primary types and the Tunisian salt tolerant cv. OR (**Figure 4**). Values of DW secondary RLs largely exceeded those of BW introgression lines, again supporting the hypothesis of a positive 7el_1_L + 7EL interaction in promoting root and leaf growth even under highly stressful conditions. On the other hand, when two RLs (R112+ and R74-10/R112+) and their CLs were exposed to a few days salt “shock” starting from germination, R112+ had a significant advantage over its CL for both root and leaf length, and over R74-10/R112+ for LL at 100 mM NaCl, before both root and leaf length sharply declined to similar values in all lines (**Table 1**).

At cellular level plant growth depends on cell proliferation through the mitotic cycle and subsequent cell expansion. Progression through the cycle, especially at the level of the G1/S and G2/M checkpoints, is controlled by a wide array of regulatory mechanisms, including the activity of cyclins and cyclin-dependent kinases (CDKs), as well as epigenetic changes-driven chromatin remodeling, and shows a variety of even conspicuous alterations in response to abiotic stresses (Zhao et al., 2014; Qi and Zhang, 2020; Kamal et al., 2021). In Arabidopsis, salt stress caused a severe disruption of mitotic activity, attributed to a block at the G2/M transition (West et al., 2004). In maize, heat and cold treatments induced a pronounced cell accumulation at the G2/M transition, NaCl treatment resulted in extensive inhibition of both S and G2/M phases, while drought stress caused most of the cells to be blocked in G1 (Zhao et al., 2014). In a later study (Kamal et al., 2021), both salt and drought stresses were found to arrest cell cycle in S phase. In wheat, knowledge of the effect of abiotic stress on cell cycle progression is very scarce. In seedlings subjected to mild water stress, leaf elongation rate and mitotic activity were reduced in mesophyll cells, due to a slowed progress from the G1 to the G2 phase and decreased activity of CDKA, required for entry into mitosis (Schuppler et al., 1998). Overall, a similar situation was observed in the present study, where, compared to the unstressed condition, exposure to salt stress led to higher percentage of nuclei in G1, of higher degree in BW than in DW lines, but in all instances independent of the genotype (similar values in introgression and in wheat-only lines, see **Table 2**). In this frame, the stress impact on the subsequent S phase, corresponding to a lower proportion of cells than in the unstressed condition, can be considered an expected consequence. Among the present materials, BW lines had a higher reduction (>50%), with no apparent effect of presence/absence of any *Thinopyrum* introgression (**Table 2B**). Instead, the behavior of DW lines was less homogeneous: RLs R112+ and R69-9/R5+, with similar percent reduction to cv. OR, had a more conspicuous decrease than their CLs (particularly evident in R112+ versus. R112–), suggestive of a specific influence of the alien introgression(s) on the stress response (**Table 2A**). However, an opposite trend was detected in R74-10/R112, exhibiting a lower decrease of S-phase nuclei in the RL (+) than in its CL (–) in the comparison between stressed and unstressed condition. Furthermore, the stress-induced alteration involving the G2 phase consisted of an increment (12%–13%) in RLs R112+ and R69-9/R5+ (and so in cv. OR), while a similar variation but in opposite direction was exhibited by R74-10/R112+ and by all BW lines. At present, no sufficiently substantiated explanation can be provided to account for the observed differences among genotypes in relation to their performance toward salinity stress. However, the overall behavior of materials analyzed here is in line with the early considerations by West et al. (2004), based on their work in Arabidopsis, and those of subsequent literature on various stresses and species (see above), suggesting that a block in cell cycle progression is rapidly induced by stress exposure. This would prevent entry into stages where cells are more vulnerable to damage (e.g. S or M-phase) and allow the cellular defense system to be activated, before progressing again at default rates once the plant has adapted to the stress.

Among the early adaptive mechanisms is the control of water uptake, disrupted by the early occurring osmotic stress due to salt increase around the roots. This, through immediate reduction of stomatal conductance, represents the initial and most profound cause of decline in CO_2_ assimilation rate and hence photosynthesis (Munns, 1993, 2002; James et al., 2008; Carillo et al., 2011; Hu and Schmidhalter, 2023). In all DW RLs, a better plant growth under salt stress was accompanied by a less compromised water status (higher RWC) than in CLs, with secondary RLs and corresponding primary types (R5+ and R112+) showing significantly higher values than cv. OR at the highest NaCl concentration. Indeed, in two of the secondary RLs (R69-9/R5+ and R69-9/R112+), no water stress was evident at 100 mM salt, and the same lines maintained the highest RWC absolute values at 200 mM. Co-presence of 7el_1_L and 7EL, particularly in R69-9/R112+ and R74-10/R112+, also contributed to limit the stress-induced decrease of photosynthetic pigments (both total chlorophylls and carotenoids, see **Figure 5**), with no major difference between the two salt stress conditions applied. Maintenance or, more, increase of carotenoids, acting as auxiliary light-collecting pigments and protectors of photosynthetic apparatus, seems to be a distinctive feature of halophytes compared with glycophytes (Bose et al., 2014). Here, the complete 7E chromosome from *Th. elongatum*, as in the CS7E(7D) substitution line, was not apparently contributing to maintenance of photosynthetic pigment content under stress, while a more positive effect was exerted by *Th. ponticum* 7el_1_ in CSTr#12. A similar 7el_1_ effect was not expressed by DW primary RLs (except for somewhat better values in R5+ at 100 mM salt), although R112+, among them, had shown high photosynthetic efficiency under normal field conditions (Kuzmanović et al., 2016) and induced heat stress (Giovenali et al., 2023).

### Na^+^ and K^+^ ion accumulation and compatible solutes

In addition to buffering the changes in water relations (osmotic tolerance), common to plants subjected to a water stress (e.g. Munns, 2002), a specific and essential salt tolerance mechanism takes place in a later phase and consists in the ability to minimize ion accumulation, mainly of Na^+^, particularly in transpiring leaves. In its absence, ions build up rapidly in cell walls, leading to cell dehydration, and in the cytoplasm, impairing physiological functions such as photosynthesis as well as protein synthesis and enzyme activity, while boosting ROS generation (e.g., Munns, 2002; Munns et al., 2006; Carillo et al., 2011). For many such cell functions, of both basic metabolism and salt stress-defense related, not only low Na^+^ content, but also adequate levels of K^+^ are essential (Wu et al., 2018b). In all mechanisms underlying control of ion concentration in the various tissues (from Na^+^ extrusion in the RE zone and vacuolar Na^+^ sequestration ability in the mature root zone and hence its limited transfer to shoot and leaves, up to K^+^/Na^+^ selectivity and K^+^ retention ability in the root and leaf mesophyll, thus maintaining functional K^+^/Na^+^ ratios), DW proved to be less efficient than BW (Wu et al., 2014; Yang et al., 2014; Wu et al., 2018a; Wu et al., 2018b). In fact, all CLs of DW RLs, lacking any *Thinopyrum* introgression, showed much higher (almost doubled) Na^+^ accumulation in their leaves than BW cv. CS, particularly at 200 mM salt (**Figure 10C**). However, in the presence of either 7el_1_ or 7el_1_+7E segments, in all DW RLs, and so in the tolerant cv. OR, Na^+^ sharply decreased (by around 70% at 200 mM NaCl), reaching approximately equivalent amounts to those of the BW introgression lines at both salt concentrations. A similar trend was observed in roots, although in this case presence of any *Thinopyrum* introgression seemed somewhat more effective at the BW level and did not represent a major discriminating factor between RLs and CLs, as shown by the PCA analysis (**Figure 11**). An accompanying effect of *Thinopyrum* transfers concerned K^+^ concentrations in root and leaf tissues. Of this critical ion for several functions associated with tolerance to salinity and other stresses, including a signaling role in various adaptive responses, regulation of cell cycle progression and accumulation of water-soluble carbohydrates (Wu et al., 2018b), DW RLs retained over 20% higher amounts than BW introgression lines, both in roots and leaves (**Figures 10E, H**), and exceeded as well the DW cv. OR, at least under moderate salinity stress.

Whereas beneficial factors were provided by the segmental introgressions of halophytic origin which minimized entry and cytosolic concentration of salt into juvenile plants of DW-*Thinopyrum* spp. RLs, not less effective was their impact on tolerance mechanisms (collectively referred to as “tissue tolerance”; Munns et al., 2006; Munns and Tester, 2008), usually activated to contrast the effects of salt that inevitably gets in. Among these, is the increased production of compatible solutes, which have a fundamental role in balancing the altered cell osmotic pressure following early water stress (see above) and, together with K^+^, also intracellular toxic ion compartmentalization. The amounts of both proline and TSS accumulated in the leaf tissue of DW secondary RLs largely exceeded those of primary types (and of respective CLs), besides that of cv. OR, especially at 200 mM NaCl. No major contribution to proline accumulation appeared to be due to the *Th. ponticum* 7el_1_L segments, as, indeed, previously observed in response to heat stress (Giovenali et al., 2023). Thus, the observed higher production seems to be entirely due to the 7EL effect. The trend was similar for TSS, more abundantly built up in leaves of 7el_1_+7E “nested” recombinants compared with 7el_1_ types and cv. OR subjected to the highest salt concentration. Particularly for TSS, the more severe stress condition was apparently the one specifically inducing this protection mechanism, not significantly differentiating the DW and BW genotypes’ response under a less intense stress (**Figure 7**). One reason for this may reside in the high metabolic cost associated with synthesis of organic solutes (Munns, 2002; Munns et al., 2006; Munns and Tester, 2008).

### Oxidative stress effects and antioxidant response

That cell structures of both DW and BW lines benefited for their integrity and functioning from presence of the alien introgressions, was proven by the lower membrane damage (as from lower MDA content) of all wheat-*Thinopyrum* spp. lines, showing similar or lower values than cv. OR, and significantly lower than their “wheat-only” CLs (**Figures 7A, B**). In BW, the effect of 7el_1_ (CSTr#12) and 7E in reducing MDA content versus CS was of comparable magnitude, and so it was, overall, among DW RLs. This indicates the absence of a clear-cut additivity between factors of either *Thinopyrum* derivation, if not some more prominent effect of 7el_1_L segments (R5+, R112+, and R23+ RLs) at 200 mM salt (**Figure 7A**). Limited membrane lipid peroxidation is one important indicator of an efficient antioxidant system, able to contrast the deleterious effects of excessive ROS production. Additional evidence of a stronger antioxidant defense of the wheat-*Thinopyrum* materials than their controls came from lower amount of hydrogen peroxide (H_2_O_2_), the most stable of ROS, thus the only one that can diffuse to adjacent subcellular compartments and cross neighboring cells (e.g., Pandey et al., 2017). At 200 mM salt, H_2_O_2_ was reduced by 25% in BW introgressions versus normal CS, and in most DW RLs showed a 40% (R112+, R69-9/R112+) or even higher (R74-10/R112+) decrease versus their controls (**Figures 7C, D**).

To bring about effective scavenging of excessive ROS accumulation, the activity of enzymatic and non-enzymatic molecules with antioxidant properties was enhanced by the presence of *Thinopyrum* spp. introgressions (**Figures 8**, **9**). A large body of evidence demonstrates a higher activity, either constitutive and/or salt-stress induced, of antioxidant enzymes in halophytic versus glycophytic species of the same or closely related plant genera (Bose et al., 2014). In *Th. ponticum* (Tong et al., 2022) and *Th. elongatum* (Sheikh-Mohamadi et al., 2022) lines/ecotypes, screened for potential cultivation in saline zones of Iran and China, respectively, and so in a *Tritipyrum* (BW-*Th. elongatum*) amphiploid (Peng et al., 2022), the best morpho-physiological and agronomic performance was always associated with the highest increase of enzymatic and non-enzymatic antioxidant activities. In the present materials, salt stress induced the most prominent activity of SOD, CAT, POD, and APX enzymes in R112+ among the DW primary RLs and in the three secondary RLs, particularly the R112+ derivatives (R69-9/R112+ and R74-10/R112+), all of them exceeding their CLs and the tolerant cv. OR, as confirmed by the PCA as well (**Figure 11**). This evidence suggests that main promoting factors might reside in the 7el_1_L portion common to the mentioned RLs (R112-specific), and the somewhat better performance of CSTr#12 than CS7E(7D) versus normal CS (at least for POD and APX at 200 mM NaCl) seems to confirm this hypothesis. Nonetheless, a further contribution from presence of 7EL segments, which would explain the highly positive enzymes values of R69-9/R5+ and the highest activity peaks often displayed by R69-9/R112+ and R74-10/R112+ (**Figure 8**), cannot be excluded.

For a strong antioxidant activity, the concomitant increase of CAT and APX is certainly relevant, in view of their functional cooperation in H_2_O_2_ detoxification, although the most crucial role of APX is indisputable (see, e.g., Mizuno et al., 1998; Caverzan et al., 2012; Sofo et al., 2015; Pandey et al., 2017; Haider et al., 2021). In fact, APX (various isoforms) is a key enzyme for maintenance of the cell redox balance in all living organisms, functioning, among other things, as a linking molecule in the AsA–GSH cycle. In the latter, APX uses ascorbate, the physiologically active form of AsA, as specific electron donor to convert H_2_O_2_ into H_2_O, while AsA and GSH pools are maintained in different cell compartments (Sofo et al., 2015; Pandey et al., 2017; Hasanuzzaman et al., 2019). Together with GSH, AsA is one of the universal non-enzymatic antioxidants involved not only in ROS scavenging, with high AsA levels being essential to keep the labile APX isoenzymes in full operation (Caverzan et al., 2012), but also in modulating several fundamental functions in plants, both under stress and non-stress conditions (Akram et al., 2017). Salt stress was found to decrease ascorbate content in wheat at the vegetative and reproductive stage, although to a minor extent in tolerant versus susceptible genotypes (Sairam et al., 2005; Athar et al., 2008; Feki et al., 2017). This was also the case for the present materials, all wheat-*Thinopyrum* spp. lines exhibiting a significantly lower reduction than lines devoid of alien introgressions (**Figure 9**). In this frame, it is particularly noteworthy the performance of the three DW secondary RLs: not only they had the highest absolute ascorbate content in control conditions (0 mM NaCl) but also they were the only tolerant lines (including BW introgressions and DW cv. OR) that maintained it almost unchanged at 100 mM, before it declined to comparable values in all introgression lines and in cv. OR at 200 mM NaCl.

To make DW secondary RLs top performers in several aspects of the antioxidant and overall tolerant response to salinity stress, one of the likely multiple promoting factors of both 7el_1_L and 7EL origin could reside in the peculiar gene content of their *Th. elongatum* introgression. Within the distal end of the 7EL arm, a gene encoding a GST type, not present in plants, was detected, which some accessions of *Thinopyrum* and related perennial species (though not the *Th. ponticum* accession possessing 7el_1_L) acquired from an endophytic fungal species via horizontal transfer (Wang et al., 2020; Guo et al., 2022). This gene was considered a likely candidate for the Fusarium resistance phenotype of BW-*Thinopyrum* introgression lines and identified with the *Fhb7* locus (Wang et al., 2020; Konkin et al., 2022). GSTs, a multigene family of isozymes that catalyze the conjugation of GSH to a wide array of electrophilic and hydrophobic substrates, are known to be responsive to a multitude of biotic and abiotic stressors (Kumar and Trivedi, 2018; Estévez and Hernández, 2020). In addition to quenching reactive and harmful molecules with the addition of GSH, certain GST types take part in the AsA-GSH pathway by regenerating ascorbate at the expense of GSH (Dixon et al., 2002; Whitbread et al., 2005). A recent analysis of the metabolome of the *Fusarium* spp. resistant DW secondary RL R69-9/R5+, carrier of *Fhb7E* locus (*Fhb7* specific to *Th. elongatum* 7E, Kuzmanović et al., 2019) and of its near-isogenic CL (no *Thinopyrum* introgression) clearly highlighted the former to constitutively express and more efficiently activate upon pathogen exposure a significantly more complex matrix of defense pathways and specific metabolites than its CL, including GSH (Fanelli et al., 2023). While a possible enhancing contribution of the GST of fungal origin to the antioxidant response of R69-9/R5+ was taken into consideration, a compound nature was hypothesized for the *Fhb7E* locus, with additional 7EL genes flanking the fungal *GST* and supporting the overall resistance function (Fanelli et al., 2023). This view, which would account for the wide array of metabolites and pathways differentiating the *Fhb7E*+ from the *Fhb7E*– response (Fanelli et al., 2023), might also apply to the present investigation, where several of the identified response mechanisms against a different stressor (NaCl), notably those contrasting the oxidative burst caused by ROS, are known to be similarly activated (e.g., Pandey et al., 2017; Ramegowda et al., 2020).

Besides the 7EL contribution, the important aid of 7el_1_L-derived factors, particularly associated with part of the R112-specific segment, emerged for several parameters expressed by the DW secondary RLs analyzed here (see above). This confirms the view that transfer of a suite of genes, rather than single genes, as in the finely engineered lines employed in this work, may well be the way forward to effectively impact on salinity tolerance of glycophytes by use of halophytic germplasm (Flowers and Yeo, 1995; Flowers and Colmer, 2015; Rawat et al., 2022). Remarkably, the 7el_1_L+7EL “nested” introgressions can be easily transferred into BW by recombination in the shared homologous 7A regions in DW × BW crosses.

Considering the common ancestry and cytogenetic affinity relating *Triticum* and *Thinopyrum* species, largely demonstrated for group 7 chromosomes (e.g., Ceoloni et al., 2014), one can assume that in this, as in similar comparisons between halophytes and allied glycophytes, differences in tolerance are predominantly due to the greater robustness of the employed mechanisms in the former, rather than to qualitative differences (Flowers and Colmer, 2008, 2015; Volkov, 2015; Isayenkov and Maathuis, 2019). Plausibly, apart from the exceptional case of the *GST* gene of exotic origin described above, different thresholds toward salinity-induced injuries are associated with different *Triticum* versus *Thinopyrum* homoeoalleles, which made our chromosomally engineered products much better responding to the imposed stress. While comparative genomic and transcriptomic investigations are currently underway on the DW-*Thinopyrum* spp. near-isogenic RLs to elucidate these aspects, an important result of practical value is the excellent performance of several of them in field trials recently carried out in an extremely stressful Algerian environment. In the experimental site of Biskra (South of Saharan Atlas), characterized by very arid and saline soil conditions (EC 11-15 ds/m; see, e.g., Singh, 2022), further exacerbated by indispensable irrigation (EC 16-20 ds/m in irrigated plots), top yielder for three consecutive seasons (2019–2022) turned out to be the R69-9/R5+ RL, followed by R69-9/R112+ and then R5+ (Kuzmanović et al., 2022).

In conclusion, the promising outcomes of the work described here, help pave the way to sustainably achieving the ambitious goal of growing wheat, even the less tolerant DW, on saline soils that the crop will have to increasingly cope with (Shahzad et al., 2013; Miransari and Smith, 2019; Rawat et al., 2022).

## Data availability statement

The original contributions presented in the study are included in the article/**Supplementary Material**. Further inquiries can be directed to the corresponding authors.

## Author contributions

ST: Writing – review & editing, Writing – original draft, Visualization, Investigation, Formal Analysis, Data curation. DG: Writing – review & editing, Writing – original draft, Visualization, Methodology, Investigation, Data curation, Conceptualization. LK: Visualization, Validation, Formal Analysis, Data curation, Writing – review & editing, Resources, Conceptualization. OJ: Validation, Investigation, Writing – review & editing. AF: Visualization, Investigation, Writing – review & editing. AC: Visualization, Data curation, Writing – review & editing. RBA: Validation, Formal Analysis, Writing – review & editing. FB: Methodology, Writing – review & editing, Writing – original draft, Resources, Project administration, Funding acquisition, Conceptualization. CC: Writing – review & editing, Writing – original draft, Supervision, Resources, Project administration, Funding acquisition, Conceptualization.
