# Supplementary material for: Coping with salinity stress: segmental group 7 chromosome introgressions from halophytic Thinopyrum species greatly enhance tolerance of recipient durum wheat
Source: Front Plant Sci. 2024 Apr 29;15:1378186. doi: 10.3389/fpls.2024.1378186 (PMC11099908; doi:10.3389/fpls.2024.1378186)

Table S1. ANOVA mean squares for morpho-physiological and biochemical traits.

	DW			BW		
**Factors**	T	G	T x G	T	G	T x G
**df**	2	12	24	2	4	2
**Trait**						
**SG**	22095.73***	56.63***	10.38***	2195.90***	20.95***	24.37***
**RE**	124.16***	18.32***	0.68***	43.63***	1.15***	0.22***
**LSA**	11.82***	0.23***	0.04***	2.59***	0.07***	0.02***
**RWC**	2373.40***	90.84***	19.48***	448.20***	28.14***	4.85***
**Chl**	54.39***	2.36***	0.49***	9.98***	0.14***	0.01***
**Car**	0.48***	0.02***	0.003***	0.06***	0.005***	$8.017\ 10^{-5}$***
**Pro**	119974.59***	6815.78***	1225.80***	21479.76***	447.95***	2.34***
**TSS**	6690.50***	229.00***	58.75***	1068.00***	39.32***	5.62***
**MDA**	756.31***	67.89***	18.43***	120.61***	18.76***	17.64***
**H_2O_2**	177.69***	7.15***	1.87***	15.96***	2.30***	1.03***
**SOD**	4087.48***	137.64***	32.81***	505.58***	48.30***	18.66***
**CAT**	2203.49***	45.06***	13.68***	357.46***	12.59***	5.38***
**POD**	3370.43***	118.85***	30.49***	456.43***	20.22***	8.66***
**APX**	504.32***	30.66***	7.15***	43.94***	16.05***	5.31***
**Asc**	409.14***	151.58***	2.96***	45.79***	41.33***	2.52***
**Na^+ roots**	5326859.62***	69053.62***	20260.03***	799935.27***	47370.32***	39058.54***
**K^+ roots**	2099687.53***	23982.79***	12000.40***	415365.39***	22395.53***	12593.27***
**Na^+ leaves**	7837770.77***	507261.58***	210111.67***	705137.05***	48260.37***	47312.84***
**K^+ leaves**	1232525.43***	16562.63***	9162.84***	21038.93***	5183.59***	3819.62***

*DW = durum wheat lines; BW = bread wheat lines. df, degrees of freedom; G, genotype; T, treatment; T x G = treatment x genotype interactions. *** indicate significance at $p < 0.001$ level*

Figure S1. Modelling of cell cycle phases in the R112+ durum wheat-*Thinopyrum ponticum* recombinant line and its R112– control line at 0 mM and 200 mM NaCl by Kaluza software (Beckman Coulter, v.2.1).

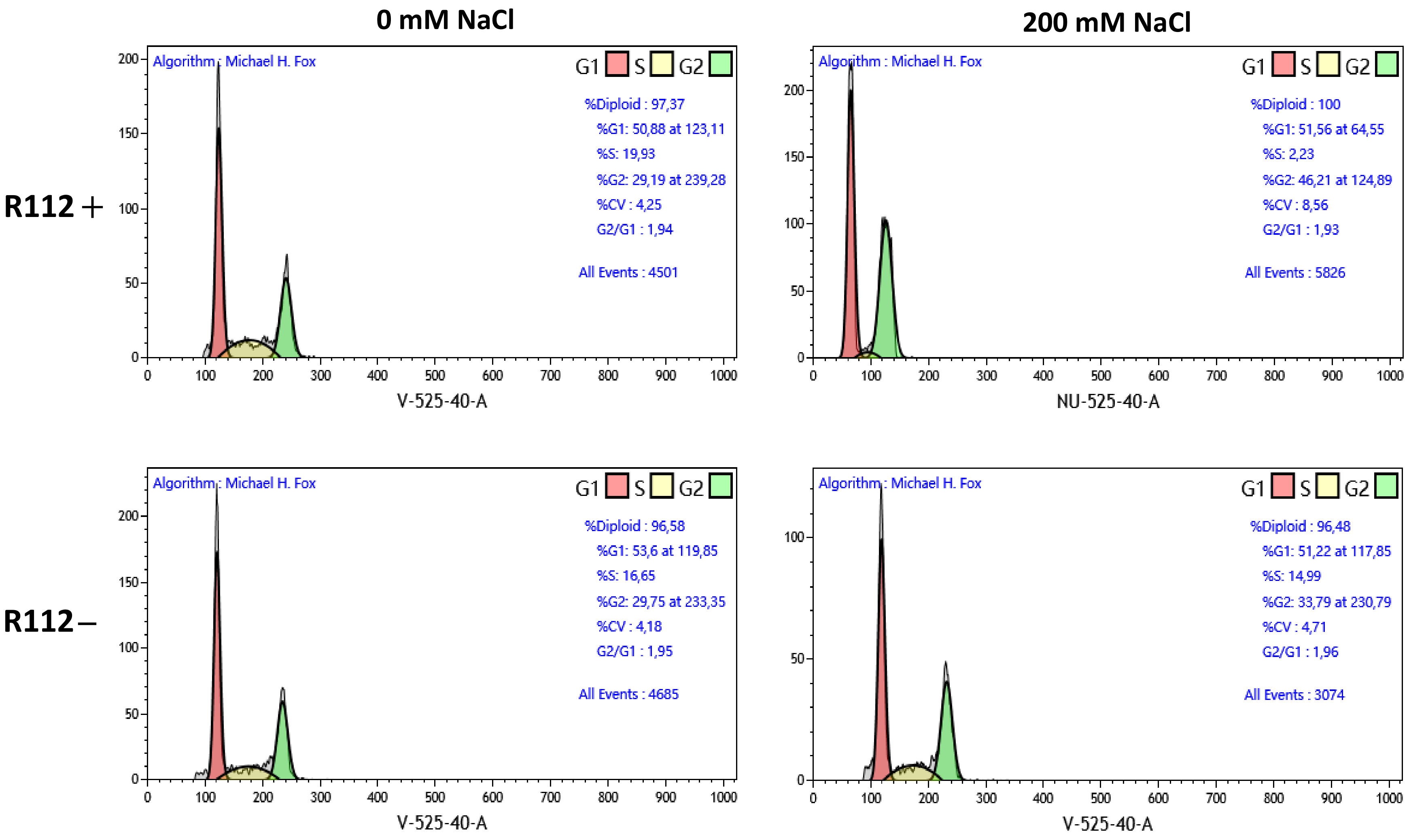

Supplement: Supplementary file 1 [file DataSheet_1.pdf]
